# Concurrent and longitudinal neurostructural correlates of irritability in children

**DOI:** 10.1038/s41386-024-01966-4

**Published:** 2024-08-17

**Authors:** Camille Archer, Hee Jung Jeong, Gabrielle E. Reimann, E. Leighton Durham, Tyler M. Moore, Shuti Wang, Devisi A. Ashar, Antonia N. Kaczkurkin

**Affiliations:** 1 Department of Psychology, Vanderbilt University, Nashville, TN USA; 2 Department of Psychiatry, Perelman School of Medicine, University of Pennsylvania, Philadelphia, PA USA

**Keywords:** Human behaviour, Predictive markers

## Abstract

Irritability, or an increased proneness to frustration and anger, is common in youth; however, few studies have examined neurostructural correlates of irritability in children. The purpose of the current study was to examine concurrent and longitudinal associations between brain structure and irritability in a large sample of 9–10-year-old children. Participants included 10,647 children from the Adolescent Brain Cognitive Development^sm^ Study (ABCD Study^®^). We related a latent irritability factor to gray matter volume, cortical thickness, and surface area in 68 cortical regions and to gray matter volume in 19 subcortical regions using structural equation modeling. Multiple comparisons were adjusted for using the false discovery rate (FDR). After controlling for age, sex, race/ethnicity, scanner model, parent’s highest level of education, medication use, and total intracranial volume, irritability was associated with smaller volumes in primarily temporal and parietal regions at baseline. Longitudinal analyses showed that baseline gray matter volume did not predict irritability symptoms at the 3rd-year follow-up. No significant associations were found for cortical thickness or surface area. The current study demonstrates inverse associations between irritability and volume in regions implicated in emotional processing/social cognition, attention allocation, and movement/perception. We advance prior research by demonstrating that neurostructural differences associated with irritability are already apparent by age 9–10 years, extending this work to children and supporting theories positing socioemotional deficits as a key feature of irritability.

## Introduction

Irritability, characterized by heightened susceptibility to frustration and anger, is a transdiagnostic symptom frequently noted as a primary reason for a child’s referral to mental health services [1–3]. Recent research has highlighted the complex interplay between neurostructural abnormalities and the manifestation of irritability in children. Specifically, irritability may be the result of disruptions in neurocircuits that contribute to attentional bias toward negative stimuli or the negative interpretation of neutral stimuli [3–6]. Others have implicated differences in sensory processing, with those with irritability showing heightened sensitivity to environmental stimuli, such as noise, light, or tactile sensations [7–9]. Alternatively, irritability may be due to differences in memory-related brain regions, which could lead to enhanced retrieval of negative associations and expectations of the unpleasant nature of encountering the perceived irritant [10, 11]. Finally, irritability could result from increased engagement of self-referential neurocircuitry, where the individual’s subjective experience is centered on personal distress, amplifying the perception of irritability [6, 12, 13]. Despite evidence of the importance of irritability symptoms in predicting concurrent and future mental health problems [3, 14, 15], there is a lack of research examining the neurobiological mechanisms underlying irritability in children, especially for brain structure. Additionally, work in this area has not determined whether these neural correlates of irritability are distinct from, or rather a component of, broader psychopathology. Thus, research is needed that examines neurostructural differences associated with irritability symptoms in younger samples.

Across multiple cross-sectional studies, differences in gray matter volume, cortical thickness, and surface area have been associated with irritability; however, there remain discrepancies in terms of the areas implicated. Studies focusing on gray matter volume show that irritability in youth is associated with smaller volumes in the putamen, internal capsule, frontal areas, and cingulate [16–19]. Other research instead demonstrates an association with larger volumes in the cingulate, in addition to larger hippocampal, insula, and medial orbitofrontal regions [19]. However, the sample in the Dennis et al. [19] study was unique in that they recruited youth specifically for exposure to early life stress. In terms of cortical thickness, higher endorsement of irritability has been associated with thinner cortices in temporal regions and the superior frontal gyrus [16, 20]. One study found that irritability was associated with less surface area in the bilateral rostral prefrontal cortex and the precuneus [21]. There are also studies demonstrating no significant relationship between irritability symptoms and regional gray matter volume, cortical thickness, or surface area [22–24]. Since these studies span large age ranges from childhood to adulthood, discrepant findings may be due to complex changes across development.

Longitudinal research further shows that irritability is associated with changes in brain structure over time. One study found that higher school-age irritability was associated with smaller gray matter volume in regions associated with emotion in pre-adolescence and with larger gray matter volume in areas such as the cerebellum [25]. Other studies show that higher irritability was associated with larger volumes and less volume contraction in brain regions that tend to decrease in volume over the developmental period [19]. In terms of cortical thickness, elevated irritability is associated with cortical thinning in brain regions implicated in emotion regulation [20]. Pagliaccio et al. identified distinct trajectories of irritability symptoms and found three classes of longitudinal irritability—elevated irritability that decreases over time, consistently low irritability, and consistently elevated irritability—with those exhibiting consistently elevated irritability showing a thicker cortex in the left superior frontal and temporal gyri and the right inferior parietal lobule [26]. Divergent longitudinal findings in the literature may be due to relatively small sample sizes and analyzing brain differences across a wide age range, highlighting the need for research focused on distinct periods of development.

While some neurostructural correlates of irritability have been identified, it remains unclear whether these associations are specific to irritability or indicative of mental health symptoms more generally. Research suggests that many mental health disorders, especially anxiety and depression, share common features such as negative affect or neuroticism [27, 28]. Additionally, a broad range of mental health conditions are characterized by similarities in brain activation patterns [29, 30] and impaired inhibitory control [31–33], particularly in regions associated with processing and assigning salience to environmental stimuli, such as the salience network [34]. The neurostructural and functional alterations observed across various mental health disorders highlight their central role in general psychopathology. Given that irritability is one component of negative affect and neuroticism [35, 36], the neurostructural substrates of irritability may simply reflect associations with broader mental health symptoms. A number of studies examining structural associations with negative affect/neuroticism have identified many regions that are similar to those found in the irritability literature [37, 38]. Thus, it is important to investigate whether these neurostructural differences are specific to irritability or reflect an underlying component of psychopathology more generally.

Most studies examining neural correlates of irritability in youth have relied on relatively modest sample sizes, have used wide age ranges which can obscure developmental changes, or have focused on adolescence specifically. Given that childhood irritability serves as a significant risk factor for developing future anxiety and depressive disorders [14], it is important to investigate neural substrates of irritability in children and determine whether structural differences are associated with later symptoms. Moreover, it is important to establish whether these neurostructural differences are unique to irritability or reflect a broader aspect of psychopathology. Therefore, the purpose of the current study was to examine the neural correlates of irritability in 9–10-year-old children with a focus on identifying structural differences that may be specific to irritability symptoms. To accomplish this, we utilized data from 11,868 children from the Adolescent Brain Cognitive Development (ABCD) Study to examine associations at 9–10 years of age and over the next 3 years [39]. Using confirmatory factor analysis, we derived a latent variable of irritability and related this to gray matter volume, cortical thickness, and surface area, both with and without controlling for anxiety symptoms, depressive symptoms, or total mental health symptoms. Given the divergent findings in the literature, we conducted an exploratory whole-brain analysis as we had no specific hypotheses regarding which brain regions would be associated with irritability.

## Methods and materials

### Participants

The present analyses used the baseline and 3rd-year follow-up data from release 5.1 of the ABCD Study [39]. The use of this de-identified dataset was approved by the institutional review board at Vanderbilt University. Participants in the ABCD Study were recruited at 21 sites across the United States [39, 40]. The baseline time point of the ABCD Study includes data from 11,868 children between 9 and 10 years of age. After excluding cases with missing data or who failed to pass quality assurance measures, the final sample size included in analyses was *N* = 10,647. A summary of the demographic characteristics of the sample used in analyses can be found in Table 1. For details on the generalizability of the ABCD Study sample, see the Supplementary Information.

**Table 1:** Demographic characteristics of the sample included in analyses of associations between irritability and brain structure (*N* = 10,647).

	Mean	SD
Age (years)	9.9	0.63

	*N*	%
Gender		
Female	5113	48.02
Male	5534	51.98
Race-ethnicity		
White	5596	52.56
Hispanic	2187	20.54
African American	1534	14.41
Other	1330	12.49
Household annual income		
<$5000	351	3.30
$5000–$11,999	372	3.49
$12,000–$15,999	253	2.38
$16,000–$24,999	464	4.36
$25,000–$34,999	580	5.45
$35,000–$49,999	820	7.70
$50,000–$74,999	1344	12.62
$75,000–$99,999	1441	13.53
$100,000–$199,999	3002	28.20
≥$200,000	1128	10.59
Missing	892	8.38
Parental education		
No degree	535	5.02
Highschool degree/GED	1275	11.98
Some college	1742	16.36
Associate’s degree	1376	12.92
Bachelor’s degree	3033	28.49
Master’s degree	2050	19.25
Professional/doctoral degree	636	5.97

The “Other” race-ethnicity category includes those who were identified by their parent as American Indian/Native American, Alaska Native, Native Hawaiian, Guamanian, Samoan, Other Pacific Islander, Asian Indian, Chinese, Filipino, Japanese, Korean, Vietnamese, Other Asian, or other race.

### Deriving a latent measure of irritability

**Fig. 1:**
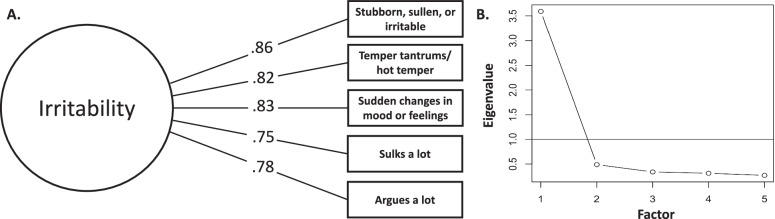
Creating a latent irritability variable. **A** We used confirmatory factor analysis to understand how five irritability items from the Child Behavior Checklist relate to each other. The standardized loadings (the coefficients that represent the strength of the relationship between observed variables and the underlying latent factor) are provided. The results showed that this model fits the data well. **B** The scree plot shows the number of possible factors on the *x*-axis and the eigenvalues (which indicate how much variance each factor explains) on the *y*-axis. The dashed line at an eigenvalue of 1 represents a standard cutoff point. If a factor’s eigenvalue is 1 or higher, it is generally retained in the model. The scree plot indicates a unidimensional measure of irritability.

Irritability was measured using the Child Behavior Checklist (CBCL) [41]. The CBCL is a parent-report measure that contains 119 items describing behaviors and emotions in children. Items were rated on a 3-point scale (0 = *not true (as far as you know)*, 1 = *somewhat or sometimes true*, and 2 = *very true or often true*). Irritability was defined as a latent variable comprised of five items from the CBCL, as has been done previously [42]. Standardized loadings and a scree plot are shown in Fig. 1a, b. Of note, prior work on irritability has examined two approaches for defining irritability: five CBCL items or the five CBCL items plus three items from the Kiddie Schedule for Affective Disorders and Schizophrenia [42]. As both approaches showed comparable internal consistency, we chose to use the five CBCL items in the present study. These five items showed good internal consistency in the current study (Cronbach’s *α* = 0.80). There was very little missing data on this measure, with 0.001% of the sample missing values on these items. For additional details on the creation of the latent irritability factor, see the Supplementary Information.

### Additional covariate subscales

To examine anxiety and depressive symptoms as separate covariates, we used the DSM-oriented CBCL subscales: Anxiety Problems (6 items measuring anxiety symptoms) and Affective Problems (14 items measuring depressive symptoms) [43]. We also created a CBCL total score by summing all CBCL items except the five items used in the irritability latent factor.

### Image acquisition, processing, and quality assurance

Image acquisition, processing, and quality assurance procedures have been previously documented [40, 44]. Additional details regarding imaging data exclusions can be found in previous work [44] and in the Supplementary Information.

### Statistical analyses

Analyses were conducted using structural equation modeling in Mplus version 8.8. We used the mean-adjusted and variance-adjusted weighted least squares estimator and pairwise deletion for missing data. Confirmatory factor analysis was used to derive a latent measure of irritability. Using this latent factor, we examined associations between irritability, gray matter volume, cortical thickness, and surface area in 68 cortical regions (34 regions in each hemisphere) derived from a surface-based atlas procedure [45]. In addition, for gray matter volume we tested 19 subcortical regions derived by an automated labeling procedure [46]. To account for known issues with the ABCD Study data, all analyses (1) incorporated clustering by family membership to account for siblings and multiple births (twins and triplets), (2) stratified based on site to account for site differences, (3) weighted the data by the post-stratification weights provided by the ABCD Study to make the sample more representative of the US population in terms of demographics, (4) weighted the data by non-participation weights to adjust for differences between the included and excluded samples as our prior work has shown that those excluded for failure to meet neuroimaging quality control differ on demographic variables [47], and (5) covaried by scanner model to control for differences between scanners. For each brain region, we investigated associations between irritability and brain structure (gray matter volume, cortical thickness, or surface area), controlling for age, sex, race/ethnicity, MRI scanner model, parent’s highest level of education as a proxy for socioeconomic status, intracranial volume (ICV), and current medication usage. Additionally, to test the specificity of our findings, we included anxious symptoms, depressive symptoms, or total psychopathology symptoms as an additional covariate. We then tested whether brain structure at baseline predicted future irritability symptoms collected at the 3rd-year follow-up session. To control for multiple testing across regions, we used the false discovery rate (*q* < 0.05) correction from the *stats* package in R version 4.2.1 (http://www.r-project.org/).

## Results

### Irritability is associated with smaller brain volumes in temporal and parietal regions

**Fig. 2:**
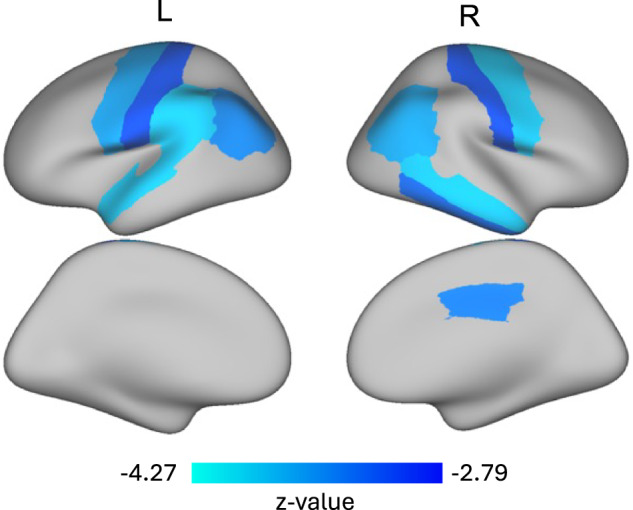
Irritability is associated with smaller gray matter volumes in primarily temporal and parietal regions. Structural equation modeling that controlled for age, sex, race/ethnicity, scanner model, medication use, parental education, and total intracranial volume revealed that greater latent irritability scores were associated with smaller volumes in the bilateral inferior parietal lobes, bilateral precentral and postcentral gyri, left superior temporal lobe, left supramarginal lobe, right inferior temporal lobe, right middle temporal lobe, and right posterior cingulate gyrus. The significant cortical regions derived from a surface-based atlas procedure [45] are shown. We utilized the false discovery rate (*q* < 0.05) to account for multiple comparisons.

Our results showed that the latent irritability factor was associated with smaller brain volumes in primarily temporal and parietal regions. Out of the 68 cortical and 19 subcortical regions examined, irritability was inversely associated with gray matter volume in 11 cortical regions, but no subcortical regions (Fig. 2; Supplementary Fig. 1; Supplementary Table 1). Some of these associations were bilateral, including the inferior parietal lobe, precentral gyrus, and postcentral gyrus. Other regions that were significantly associated with irritability included the left superior temporal lobe, left supramarginal lobe, right inferior temporal lobe, right middle temporal lobe, and right posterior cingulate gyrus. No other gray matter volume regions were significantly associated with the latent irritability factor. Next, we included anxiety symptoms, depressive symptoms, or total psychopathology symptoms as additional covariates. When anxious symptoms were included in our model, brain regions significantly associated with irritability included the left fusiform gyrus, bilateral inferior parietal lobe, bilateral precentral gyrus, left superior temporal lobe, left supramarginal lobe, right middle temporal lobe, right banks of the superior temporal sulcus, and right posterior cingulate gyrus (Supplementary Table 2). When depressive symptoms were added as a covariate, the left superior temporal gyrus, right inferior parietal gyrus, right middle temporal gyrus, and right precentral gyrus remained significantly associated with irritability (Supplementary Table 3). Finally, when we added total psychopathology symptoms as a covariate, the only region that remained significant was the right inferior parietal lobe (Supplementary Table 4). Effect sizes for these associations are shown as standardized beta estimates and *R*-squared values in all tables.

### Gray matter volume does not predict future irritability symptoms

Given the significant association between irritability and gray matter volume at baseline, we next examined whether smaller volumes predicted irritability symptoms at a future time point. We tested regional gray matter volumes as predictors of irritability at the 3rd-year follow-up, controlling for age, sex, race/ethnicity, scanner model, parent’s highest level of education, medication use, total ICV, and baseline irritability symptoms. The results showed no significant associations between gray matter volume at baseline and future irritability symptoms (Supplementary Table 5).

### Lack of associations for cortical thickness and surface area

Finally, we repeated the above analyses for cortical thickness and surface area in the 68 cortical regions. There were no significant associations between irritability and cortical thickness (Supplementary Table 6) or surface area (Supplementary Table 7).

## Discussion

The current study investigated the associations between irritability symptoms and gray matter volume, cortical thickness, and surface area while controlling for other psychopathology symptoms in a large sample of children. Additionally, this study examined whether baseline structural measures were predictive of future irritability symptoms. Irritability was associated with smaller volumes in primarily temporal and parietal regions at baseline, consistent with previous work [16–18, 48]. The analyses controlled for known confounds including age, sex, race/ethnicity, scanner model, parental education, medication use, and total ICV. Additionally, we controlled for anxious, depressive, and general psychopathology symptoms to test the specificity of our findings. While many regions continued to be associated with irritability after controlling for anxious and depressive symptoms, the results became predominantly nonsignificant when controlling for general psychopathology. This may not be surprising as irritability is a highly transdiagnostic symptom that is apparent in many mental health disorders [8, 49], making it difficult to disentangle the relative contribution of irritability to structural differences above and beyond general psychopathology.

In terms of the longitudinal analyses, gray matter volume did not predict irritability at the 3rd-year follow-up when controlling for baseline irritability. The lack of prediction at a future time point may suggest the presence of compensatory mechanisms, such as neuroplasticity or functional reorganization [50, 51], enabling children with smaller volumes in these regions to mitigate the risk of developing future irritability symptoms. Alternatively, it is possible that a longer duration between time points is necessary to observe an association between baseline brain volume and future psychopathology outcomes. It is also possible that irritability is not stable over time, reducing the likelihood of detecting an association between brain structure and future symptoms. The variability in brain development during this period, occurring at different rates in various brain regions, could account for the lack of predictive power for gray matter volume. It is important to distinguish between association and causation in this context, as observing a statistical association between gray matter volume and irritability does not necessarily imply that changes in volume cause changes in irritability. Numerous factors, including genetic predispositions, environmental exposures, and psychosocial contexts, contribute to brain development and behavioral outcomes [52–55]. Thus, these findings should not be interpreted as causal, as the observed associations between gray matter volume and irritability are likely due to a complex interplay of multiple contributing factors. While gray matter volume was not associated with future irritability in children in this sample, there could be other mechanisms or factors that influence the development of irritability over time. Our findings highlight the complexity of the relationship between brain structure and irritability symptoms and suggest that additional research is needed to fully understand these mechanisms.

No significant associations between irritability and cortical thickness or surface area were found. This contrasts with two studies linking higher irritability to thinner cortices in ages 8–22 years [16, 20] and one study showing irritability is associated with less surface area in a sample ranging from 5 to 21 years of age [21]. However, our lack of associations is consistent with two studies that also did not find significant relationships between irritability and cortical thickness or surface area [23, 24]. Notably, the studies that do find significant associations included broad age ranges extending into adulthood, whereas the current study focused on ages 9–10 years, raising the possibility that such associations may emerge later in development. Given that volume is the product of cortical thickness and surface area, it may be initially surprising that gray matter volume shows significant results while cortical thickness and surface area do not. However, prior work has shown that volume, cortical thickness, and surface area are distinct but related facets of brain structure, each offering unique information and showing different normative developmental trajectories [56, 57]. Future work collecting data from independent samples across various age groups, spanning childhood to adulthood, will be necessary to accumulate enough data to clarify the associations between irritability and structural brain development.

The regions associated with irritability in this study were primarily involved in emotional processing and social cognition, attention allocation, and movement/perception. The left superior temporal lobe, right inferior temporal lobe, right middle temporal lobe, and bilateral inferior parietal lobes were all associated with irritability in the current study. The temporal lobes are multifunctional and are associated with the processing of emotions, emotion regulation, and social cognition [58–63]. Structural differences in these regions may in part underlie the perception of negative social interactions in children with irritability, who tend to show a hostile attribution bias or the tendency to attribute hostile intent to others’ behavior [3]. This may be targeted in clinical interventions, with work showing promising results for altering negative social biases using cognitive bias modification programs, such as positive interpretation training [64]. Notably, we did not find significant differences in frontal regions commonly associated with emotional regulation (anterior cingulate and prefrontal cortices), in contrast to prior work on irritability in specific diagnostic groups [18, 65]. Given the young age of our sample, associations between irritability and frontal brain regions may not become apparent until later in adolescence. It is also possible that functional differences in these regions might exist despite the absence of structural variations [66].

Additionally, the right inferior parietal lobe was the only brain region that was significantly associated with irritability after controlling for total psychopathology symptoms, suggesting a more specific relationship between irritability and this region. The inferior parietal lobe is associated with attention allocation [62], which is consistent with research showing that youth with irritability symptoms show attentional bias to threatening or angry faces even while controlling for confounds such as anxiety levels [4, 67], suggesting that one possible mechanism for the manifestation of irritability may be a tendency to focus on negative stimuli. Thus, the finding of differences in the inferior parietal lobes in the current study supports prior literature demonstrating that these lobes are particularly relevant to the association between irritability and reward- and threat-related neural processes [68, 69]. Relatedly, we found smaller volumes in the supramarginal gyrus, a region implicated in interoception or sensing the internal state of the body, which may be related to attention reorientation [70]. Our results also showed that irritability was associated with the posterior cingulate gyrus, a central hub in the default mode network that is related to internally directed processes and regulating the focus of attention [71] and has been implicated in studies of irritability [72]. It is possible that greater sensitivity to one’s internal thoughts and physical states (e.g., heart rate increasing, body temperature rising) coupled with an attentional bias towards negative stimuli and a tendency to attribute hostile motives to others may increase the likelihood that an individual will report feeling irritable.

The final group of regions found in the current study are related to movement and perception and include the precentral and postcentral gyri [73]. Our results are consistent with prior work in youth with irritability symptoms showing smaller gray matter volumes in motor-related regions [17, 18]. Given that irritability is a transdiagnostic symptom associated with many forms of psychopathology [1, 73, 74] and that many disorders implicate the precentral and postcentral gyrus [65, 75–78], the finding of differences in movement-related regions may be expected. These results are also consistent with prior work showing that irritability and agitation (an unpleasant state of restlessness and hyperarousal that has links with motor regions) commonly present together [79].

### Clinical implications

The transdiagnostic nature of irritability has important implications for diagnosis and treatment. Recognizing irritability as a transdiagnostic trait could potentially lead to more dimensional approaches in mental health assessment that consider irritability as a core feature across many disorders. This shift could enable tailored interventions targeting the underlying mechanisms shared by disorders [80], while also aiding in the early identification of individuals at risk for developing comorbid conditions, thus facilitating more effective prevention and intervention strategies. Existing interventions for irritability are mainly focused on symptom reduction and relapse prevention, with work demonstrating that approaches promoting resilience in both parents and children are promising for early prevention, not only for irritability but also for other comorbid symptoms as well [81]. However, randomized controlled trials in children with severe irritability are needed to assess the efficacy of potential pharmacological and psychological treatments.

### Strengths and limitations

This study’s strengths include a large sample size and a narrowly defined age range, which enables us to examine the relationship between brain structure and irritability while avoiding age-related variance. While the ABCD Study sample is fairly representative in terms of demographic and socioeconomic distribution, it is not perfectly representative. Therefore, another strength of this study was the use of post-stratification weights to adjust the sample to be more representative of the US population demographics. Additionally, while previous studies focus on one point in time, we leverage longitudinal data to examine whether neurostructural measures predict future irritability symptoms.

Limitations of the current study include the use of parent-reported irritability symptoms which account for parent perceptions but do not reflect child self-report or clinician ratings. As previous research has demonstrated, parent and child-reported irritability symptoms tend to diverge [82], thus, future work should include measures of self-reported irritability as well. The ABCD Study will include a self-report version of the CBCL (the Youth Self-Report) when the participants turn 14 years old [83], which will allow future work to examine these associations using self-reported irritability symptoms. Additionally, we defined irritability as a unidimensional construct and did not differentiate phasic (i.e., outburst) versus tonic (i.e., mood) components of irritability in our analyses. Future work could aim to assess irritability as multifaceted, either through data-driven exploration of subfactors of irritability or using measures that are able to distinguish between these aspects of irritability [84, 85]. Another limitation of our study is the exclusive focus on neurobiological factors, without incorporating environmental and other influences that also play a critical role in the development and manifestation of irritability symptoms. Given the multidimensional and context-dependent nature of irritability, future work should incorporate a more integrative approach that includes genetic, environmental, and psychosocial factors to better understand the complex predictors of irritability. Finally, the effect sizes found in our analysis of brain structure and irritability were relatively small; however, previous studies with large samples have also consistently shown small but reliable associations between brain structure and behavior [86].

## Conclusions

The current study expands on prior work by examining the neurostructural correlates of irritability in youth by using a large, well-defined sample, creating a latent measure of irritability, and leveraging a longitudinal design to predict future symptoms. Our findings underscore the importance of brain regions associated with emotional processing, social cognition, attention allocation, and movement/perception in understanding irritability symptoms in children. These results support theories positing deficits in socioemotional and attentional neural circuitry as key features of irritability and demonstrate that these neurostructural differences are apparent at 9–10 years of age. Given that structural differences in these areas are implicated in many mental health problems, the current study supports the conceptualization of irritability as a transdiagnostic trait across disorders. Taken together, the results of this study provide a deeper understanding of neurostructural substrates that may be related to multiple disorders that involve irritability as a core symptom, thereby increasing our understanding of the neurobiological mechanisms underlying diverse mental health conditions.

## Supplementary information

Supplementary Materials for Concurrent and Longitudinal Neurostructural Correlates of Irritability in Children

## Data Availability

The ABCD Study data are available through the National Institute of Mental Health Data Archive (https://nda.nih.gov/abcd).

## Code availability

Analysis scripts and a corresponding wiki explaining the analyses can be found at https://github.com/VU-BRAINS-lab/Archer_Irritability_volume.

## References

[1] Beauchaine TP, Tackett JL. Irritability as a transdiagnostic vulnerability trait: current issues and future directions. Behav Ther. 2020;51:350–64. 10.1016/J.BETH.2019.10.009. 32138943 10.1016/j.beth.2019.10.009

[2] Leibenluft E, Stoddard J. The developmental psychopathology of irritability. Dev Psychopathol. 2013;25:1473–87. 10.1017/S0954579413000722. 24342851 10.1017/S0954579413000722 PMC4476313

[3] Brotman MA, Kircanski K, Stringaris A, Pine DS, Leibenluft E. Irritability in youths: a translational model. Am J Psychiatry. 2017;174:520–32. 10.1176/appi.ajp.2016.16070839. 28103715 10.1176/appi.ajp.2016.16070839 PMC13335380

[4] Naim R, Haller SP, Linke JO, Jaffe A, Stoddard J, Jones M, et al. Context-dependent amygdala–prefrontal connectivity during the dot-probe task varies by irritability and attention bias to angry faces. Neuropsychopharmacology. 2022;47:2283–91. 10.1038/s41386-022-01307-3. 35641787 10.1038/s41386-022-01307-3 PMC9630440

[5] Hirsch E, Hulvershorn L. Neural findings in pediatric irritability. In: Roy AK, Brotman MA, Leibenluft E, editors. Irritability in pediatric psychopathology. New York, NY: Oxford University Press; 2019. p. 171–94.

[6] Zisner A, Beauchaine TP. Neural substrates of trait impulsivity, anhedonia, and irritability: mechanisms of heterotypic comorbidity between externalizing disorders and unipolar depression. Dev Psychopathol. 2016;28:1177–208. 10.1017/S0954579416000754. 27739396 10.1017/S0954579416000754

[7] Benarous X, Bury V, Lahaye H, Desrosiers L, Cohen D, Guilé JM. Sensory processing difficulties in youths with disruptive mood dysregulation disorder. Front Psychiatry. 2020;11:164. 10.3389/fpsyt.2020.00164. 32265752 10.3389/fpsyt.2020.00164 PMC7104792

[8] Toohey MJ, DiGiuseppe R. Defining and measuring irritability: construct clarification and differentiation. Clin Psychol Rev. 2017;53:93–108. 10.1016/j.cpr.2017.01.009. 28284170 10.1016/j.cpr.2017.01.009

[9] Harima Y, Miyawaki D, Goto A, Hirai K, Sakamoto S, Hama H, et al. Associations between chronic irritability and sensory processing difficulties in children and adolescents. Front Psychiatry. 2022;13:860278 10.3389/fpsyt.2022.860278. 35573381 10.3389/fpsyt.2022.860278 PMC9095987

[10] Tseng WL, Abend R, Gold AL, Brotman MA. Neural correlates of extinguished threat recall underlying the commonality between pediatric anxiety and irritability. J Affect Disord. 2021;295:920–9. 10.1016/J.JAD.2021.08.117. 34706463 10.1016/j.jad.2021.08.117 PMC8554134

[11] Gotlib IH, Traill SK, Montoya RL, Joormann J, Chang K. Attention and memory biases in the offspring of parents with bipolar disorder: indications from a pilot study. J Child Psychol Psychiatry. 2005;46:84–93. 10.1111/J.1469-7610.2004.00333.X. 15660646 10.1111/j.1469-7610.2004.00333.x

[12] Crum KI, Hwang S, Blair KS, Aloi JM, Meffert H, White SF, et al. Interaction of irritability and anxiety on emotional responding and emotion regulation: a functional MRI study. Psychol Med. 2021;51:2778–88. 10.1017/S0033291720001397. 32584213 10.1017/S0033291720001397 PMC7759590

[13] Leigh E, Lee A, Brown HM, Pisano S, Stringaris A. A prospective study of rumination and irritability in youth. J Abnorm Child Psychol. 2020;48:1581–9. 10.1007/S10802-020-00706-8/TABLES/3. 33001331 10.1007/s10802-020-00706-8 PMC7554009

[14] Copeland WE, Brotman MA, Costello EJ. Normative irritability in youth: developmental findings from the Great Smoky Mountains Study. J Am Acad Child Adolesc Psychiatry. 2015;54:635–42. 10.1016/j.jaac.2015.05.008. 26210332 10.1016/j.jaac.2015.05.008 PMC4515775

[15] Vidal-Ribas P, Brotman MA, Valdivieso I, Leibenluft E, Stringaris A. The status of irritability in psychiatry: a conceptual and quantitative review. J Am Acad Child Adolesc Psychiatry. 2016;55:556–70. 10.1016/j.jaac.2016.04.014. 27343883 10.1016/j.jaac.2016.04.014 PMC4927461

[16] Seok JW, Bajaj S, Soltis-Vaughan B, Lerdahl A, Garvey W, Bohn A, et al. Structural atrophy of the right superior frontal gyrus in adolescents with severe irritability. Hum Brain Mapp. 2021;42:4611–22. 10.1002/hbm.25571. 34288223 10.1002/hbm.25571 PMC8410540

[17] Chaarani B, Kan KJ, Mackey S, Spechler PA, Potter A, Banaschewski T, et al. Neural correlates of adolescent irritability and its comorbidity with psychiatric disorders. J Am Acad Child Adolesc Psychiatry. 2020;59:1371–9. 10.1016/j.jaac.2019.11.028. 32860907 10.1016/j.jaac.2019.11.028

[18] Mulraney M, Sciberras E, Gulenc A, Efron D, Hazell P, Silk TJ. Neural correlates of irritability in a community sample of children. J Affect Disord. 2021;292:223–6. 10.1016/j.jad.2021.05.093. 34130187 10.1016/j.jad.2021.05.093

[19] Dennis EL, Humphreys KL, King LS, Thompson PM, Gotlib IH. Irritability and brain volume in adolescents: cross-sectional and longitudinal associations. Soc Cogn Affect Neurosci. 2019;14:687–98. 10.1093/scan/nsz053. 31309969 10.1093/scan/nsz053 PMC6778832

[20] Jirsaraie RJ, Kaczkurkin AN, Rush S, Piiwia K, Adebimpe A, Bassett DS, et al. Accelerated cortical thinning within structural brain networks is associated with irritability in youth. Neuropsychopharmacology. 2019;44:2254–62. 10.1038/s41386-019-0508-3. 31476764 10.1038/s41386-019-0508-3 PMC6897907

[21] Piguet C, Mihailov A, Grigis A, Laidi C, Duchesnay E, Houenou J. Irritability is associated with decreased cortical surface area and anxiety with decreased gyrification during brain development. Front Psychiatry. 2021;12. 10.3389/FPSYT.2021.744419/FULL. 10.3389/fpsyt.2021.744419 PMC8492928 34630188

[22] Gold AL, Brotman MA, Adleman NE, Lever SN, Steuber ER, Fromm SJ, et al. Comparing brain morphometry across multiple childhood psychiatric disorders. J Am Acad Child Adolesc Psychiatry. 2016;55:1027–37. 10.1016/j.jaac.2016.08.008. 27871637 10.1016/j.jaac.2016.08.008 PMC10213704

[23] Cardinale EM, Kircanski K, Brooks J, Gold AL, Towbin KE, Pine DS, et al. Parsing neurodevelopmental features of irritability and anxiety: replication and validation of a latent variable approach. Dev Psychopathol. 2019;31:917–29. 10.1017/S095457941900035X. 31064595 10.1017/S095457941900035X PMC7439289

[24] Bajaj S, Blair KS, Bashford-Largo J, Zhang R, Mathur A, Schwartz A, et al. Network-wise surface-based morphometric insight into the cortical neural circuitry underlying irritability in adolescents. Transl Psychiatry. 2021;11:581 10.1038/s41398-021-01710-2. 34759268 10.1038/s41398-021-01710-2 PMC8581009

[25] Damme KSF, Norton ES, Briggs-Gowan MJ, Wakschlag LS, Mittal VA. Developmental patterning of irritability enhances prediction of psychopathology in pre-adolescence: improving RDoC with developmental science. J Psychopathol Clin Sci. 2022;131:556–566. 10.1101/2020.04.30.070714. 35901387 10.1037/abn0000655 PMC9439570

[26] Pagliaccio D, Pine DS, Barch DM, Luby JL, Leibenluft E. Irritability trajectories, cortical thickness, and clinical outcomes in a sample enriched for preschool depression. J Am Acad Child Adolesc Psychiatry. 2018;57:336–42. 10.1016/j.jaac.2018.02.010. 29706163 10.1016/j.jaac.2018.02.010 PMC5932635

[27] Ormel J, Jeronimus BF, Kotov R, Riese H, Bos EH, Hankin B, et al. Neuroticism and common mental disorders: meaning and utility of a complex relationship. Clin Psychol Rev. 2013;33:686–97. 10.1016/J.CPR.2013.04.003. 23702592 10.1016/j.cpr.2013.04.003 PMC4382368

[28] Griffith JW, Zinbarg RE, Craske MG, Mineka S, Rose RD, Waters AM, et al. Neuroticism as a common dimension in the internalizing disorders. Psychol Med. 2010;40:1125–36. 10.1017/S0033291709991449. 19903363 10.1017/S0033291709991449 PMC2882529

[29] Broyd SJ, Demanuele C, Debener S, Helps SK, James CJ, Sonuga-Barke EJS. Default-mode brain dysfunction in mental disorders: a systematic review. Neurosci Biobehav Rev. 2009;33:279–96. 10.1016/J.NEUBIOREV.2008.09.002. 18824195 10.1016/j.neubiorev.2008.09.002

[30] Banich MT, Smith LL, Smolker HR, Hankin BL, Silton RL, Heller W, et al. Common and specific dimensions of internalizing disorders are characterized by unique patterns of brain activity on a task of emotional cognitive control. Int J Psychophysiol. 2020;151:80–93. 10.1016/J.IJPSYCHO.2020.02.002. 32032623 10.1016/j.ijpsycho.2020.02.002 PMC9058970

[31] Kang W, Hernández SP, Rahman MS, Voigt K, Malvaso A. Inhibitory control development: a network neuroscience perspective. Front Psychol. 2022;13:651547 10.3389/FPSYG.2022.651547/BIBTEX. 36300046 10.3389/fpsyg.2022.651547 PMC9588931

[32] Yan H, Lau WKW, Eickhoff SB, Long J, Song X, Wang C, et al. Charting the neural circuits disruption in inhibitory control and its subcomponents across psychiatric disorders: a neuroimaging meta-analysis. Prog Neuropsychopharmacol Biol Psychiatry. 2022;119:110618. 10.1016/J.PNPBP.2022.110618. 36002101 10.1016/j.pnpbp.2022.110618

[33] Goschke T. Dysfunctions of decision-making and cognitive control as transdiagnostic mechanisms of mental disorders: advances, gaps, and needs in current research. Int J Methods Psychiatr Res. 2014;23:41–57. 10.1002/MPR.1410. 24375535 10.1002/mpr.1410 PMC6878557

[34] McTeague LM, Huemer J, Carreon DM, Jiang Y, Eickhoff SB, Etkin A. Identification of common neural circuit disruptions in cognitive control across psychiatric disorders. Am J Psychiatry. 2017;174:676–85. 10.1176/APPI.AJP.2017.16040400. 28320224 10.1176/appi.ajp.2017.16040400 PMC5543416

[35] Shiner RL. Negative emotionality and neuroticism from childhood through adulthood. In: McAdams DP, Shiner RL, Tackett JL, editors. Handbook of personality development. New York, NY: The Guilford Press; 2018. p. 137–52.

[36] Deveney CM, Stoddard J, Evans RL, Chavez G, Harney M, Wulff RA. On defining irritability and its relationship to affective traits and social interpretations. Pers Individ Differ. 2019;144:61–67. 10.1016/J.PAID.2019.02.031. 10.1016/j.paid.2019.02.031 PMC6512980 31097847

[37] Liu X, Lai H, Li J, Becker B, Zhao Y, Cheng B, et al. Gray matter structures associated with neuroticism: a meta-analysis of whole-brain voxel-based morphometry studies. Hum Brain Mapp. 2021;42:2706–21. 10.1002/HBM.25395. 33704850 10.1002/hbm.25395 PMC8127153

[38] Wise T, Radua J, Via E, Cardoner N, Abe O, Adams TM, et al. Common and distinct patterns of grey-matter volume alteration in major depression and bipolar disorder: evidence from voxel-based meta-analysis. Mol Psychiatry. 2016;22:1455–63. 10.1038/mp.2016.72. *2016 22:10* 27217146 10.1038/mp.2016.72 PMC5622121

[39] Volkow ND, Koob GF, Croyle RT, Bianchi DW, Gordon JA, Koroshetz WJ, et al. The conception of the ABCD study: from substance use to a broad NIH collaboration. Dev Cogn Neurosci. 2018;32:4–7. 10.1016/j.dcn.2017.10.002. 29051027 10.1016/j.dcn.2017.10.002 PMC5893417

[40] Casey BJ, Cannonier T, Conley MI, Cohen AO, Barch DM, Heitzeg MM, et al. The Adolescent Brain Cognitive Development (ABCD) Study: imaging acquisition across 21 sites. Dev Cogn Neurosci. 2018;32:43–54. 10.1016/j.dcn.2018.03.001. 29567376 10.1016/j.dcn.2018.03.001 PMC5999559

[41] Achenbach TM. The Achenbach System of Empirically Based Assessment (ASEBA): development, findings, theory, and applications. Burlington, Vermont: University of Vermont Research Center for Children, Youth, and Families; 2009.

[42] Cordova MM, Antovich DM, Ryabinin P, Neighbor C, Mooney MA, Dieckmann NF, et al. ADHD: restricted phenotypes prevalence, comorbidity, and polygenic risk sensitivity in ABCD baseline cohort. J Am Acad Child Adolesc Psychiatry. 2022;61:1273–1284. 10.1016/J.JAAC.2022.03.030. 35427730 10.1016/j.jaac.2022.03.030 PMC9677584

[43] Achenbach TM, Dumenci L, Rescorla LA. DSM-oriented and empirically based approaches to constructing scales from the same item pools. J Clin Child Adolesc Psychol. 2003;32:328–40. 10.1207/S15374424JCCP3203_02. 12881022 10.1207/S15374424JCCP3203_02

[44] Hagler DJ Jr, Hatton S, Cornejo MD, Makowski C, Fair DA, Dick AS, et al. Image processing and analysis methods for the Adolescent Brain Cognitive Development Study. Neuroimage. 2019;202:116091 10.1016/j.neuroimage.2019.116091. 31415884 10.1016/j.neuroimage.2019.116091 PMC6981278

[45] Desikan RS, Ségonne F, Fischl B, Quinn BT, Dickerson BC, Blacker D, et al. An automated labeling system for subdividing the human cerebral cortex on MRI scans into gyral based regions of interest. Neuroimage. 2006;31:968–80. 10.1016/j.neuroimage.2006.01.021. 16530430 10.1016/j.neuroimage.2006.01.021

[46] Fischl B, Salat D, Busa E, Albert M, Dieterich M, Haselgrove C. Whole brain segmentation: automated labeling of neuroanatomical structures in the human brain. Neuron. 2002;33:341–55. 10.1016/S0896-6273(02)00569-X. 11832223 10.1016/s0896-6273(02)00569-x

[47] Durham EL, Jeong HJ, Moore TM, Dupont RM, Cardenas-Iniguez C, Cui Z, et al. Association of gray matter volumes with general and specific dimensions of psychopathology in children. Neuropsychopharmacology. 2021;46:1333–9. 10.1038/s41386-020-00952-w. 33479512 10.1038/s41386-020-00952-w PMC8134562

[48] Besteher B, Squarcina L, Spalthoff R, Bellani M, Gaser C, Brambilla P, et al. Brain structural correlates of irritability: findings in a large healthy cohort. Hum Brain Mapp. 2017;38:6230–8. 10.1002/hbm.23824. 28945310 10.1002/hbm.23824 PMC6866715

[49] Leibenluft E, Allen LE, Althoff RR, Brotman MA, Burke JD, Carlson GA, et al. Irritability in youths: a critical integrative review. Am J Psychiatry. 2024;181:275–290. 10.1176/APPI.AJP.20230256. 38419494 10.1176/appi.ajp.20230256 PMC12010774

[50] Rapoport JL, Gogtay N. Brain neuroplasticity in healthy, hyperactive and psychotic children: insights from neuroimaging. Neuropsychopharmacology. 2007;33:181–97. 10.1038/sj.npp.1301553. 17851542 10.1038/sj.npp.1301553

[51] Grafman J. Conceptualizing functional neuroplasticity. J Commun Disord. 2000;33:345–56. 10.1016/S0021-9924(00)00030-7. 11001161 10.1016/s0021-9924(00)00030-7

[52] Liu S, Smit DJA, Abdellaoui A, van Wingen GA, Verweij KJH. Brain structure and function show distinct relations with genetic predispositions to mental health and cognition. Biol Psychiatry Cogn Neurosci Neuroimaging. 2023;8:300–10. 10.1016/J.BPSC.2022.08.003. 35961582 10.1016/j.bpsc.2022.08.003

[53] Grossman AW, Churchill JD, McKinney BC, Kodish IM, Otte SL, Greenough WT. Experience effects on brain development: possible contributions to psychopathology. J Child Psychol Psychiatry. 2003;44:33–63. 10.1111/1469-7610.T01-1-00102. 12553412 10.1111/1469-7610.t01-1-00102

[54] Bush NR, Wakschlag LS, LeWinn KZ, et al. Family environment, neurodevelopmental risk, and the environmental influences on child health outcomes (ECHO) initiative: looking back and moving forward. Front Psychiatry. 2020;11:504260. 10.3389/FPSYT.2020.00547/BIBTEX. 10.3389/fpsyt.2020.00547 PMC7318113 32636769

[55] Whittle S, Zhang L, Rakesh D. Environmental and neurodevelopmental contributors to youth mental illness. Neuropsychopharmacology. 2024:1–10. 10.1038/s41386-024-01926-y. 10.1038/s41386-024-01926-y PMC11526094 39030435

[56] Winkler AM, Kochunov P, Blangero J, Almasy L, Zilles K, Fox PT, et al. Cortical thickness or grey matter volume? The importance of selecting the phenotype for imaging genetics studies. Neuroimage. 2010;53:1135–46. 10.1016/J.NEUROIMAGE.2009.12.028. 20006715 10.1016/j.neuroimage.2009.12.028 PMC2891595

[57] Wierenga LM, Langen M, Oranje B, Durston S. Unique developmental trajectories of cortical thickness and surface area. Neuroimage. 2014;87:120–6. 10.1016/J.NEUROIMAGE.2013.11.010. 24246495 10.1016/j.neuroimage.2013.11.010

[58] Olson IR, McCoy D, Klobusicky E, Ross LA. Social cognition and the anterior temporal lobes: a review and theoretical framework. Soc Cogn Affect Neurosci. 2013;8:123–33. 10.1093/scan/nss119. 23051902 10.1093/scan/nss119 PMC3575728

[59] Adolphs R. The neurobiology of social cognition. Curr Opin Neurobiol. 2001;11:231–9. 10.1016/S0959-4388(00)00202-6. 11301245 10.1016/s0959-4388(00)00202-6

[60] Ross LA, Olson IR. Social cognition and the anterior temporal lobes. Neuroimage. 2010;49:3452–62. 10.1016/J.NEUROIMAGE.2009.11.012. 19931397 10.1016/j.neuroimage.2009.11.012 PMC2818559

[61] Kohn N, Eickhoff SB, Scheller M, Laird AR, Fox PT, Habel U. Neural network of cognitive emotion regulation—an ALE meta-analysis and MACM analysis. Neuroimage. 2014;87:345–55. 10.1016/J.NEUROIMAGE.2013.11.001. 24220041 10.1016/j.neuroimage.2013.11.001 PMC4801480

[62] Numssen O, Bzdok D, Hartwigsen G. Functional specialization within the inferior parietal lobes across cognitive domains. Elife. 2021;10. 10.7554/eLife.63591. 10.7554/eLife.63591 PMC7946436 33650486

[63] Bzdok D, Hartwigsen G, Reid A, Laird AR, Fox PT, Eickhoff SB. Left inferior parietal lobe engagement in social cognition and language. Neurosci Biobehav Rev. 2016;68:319–34. 10.1016/j.neubiorev.2016.02.024. 27241201 10.1016/j.neubiorev.2016.02.024 PMC5441272

[64] Hawkins KA, Cougle JR. Effects of interpretation training on hostile attribution bias and reactivity to interpersonal insult. Behav Ther. 2013;44:479–88. 10.1016/J.BETH.2013.04.005. 23768674 10.1016/j.beth.2013.04.005

[65] Wiggins JL, Brotman MA, Adleman NE, Kim P, Oakes AH, Reynolds RC, et al. Neural correlates of irritability in disruptive mood dysregulation and bipolar disorders. Am J Psychiatry. 2016;173:722–30. 10.1176/appi.ajp.2015.15060833. 26892942 10.1176/appi.ajp.2015.15060833 PMC11193882

[66] Tahmasebi AM, Davis MH, Wild CJ, Rodd JM, Hakyemez H, Abolmaesumi P, et al. Is the link between anatomical structure and function equally strong at all cognitive levels of processing? Cereb Cortex. 2012;22:1593–603. 10.1093/CERCOR/BHR205. 21893681 10.1093/cercor/bhr205

[67] Salum GA, Mogg K, Bradley BP, Stringaris A, Gadelha A, Pan PM, et al. Association between irritability and bias in attention orienting to threat in children and adolescents. J Child Psychol Psychiatry. 2017;58:595–602. 10.1111/jcpp.12659. 27782299 10.1111/jcpp.12659 PMC9891207

[68] Kircanski K, White LK, Tseng WL, Wiggins JL, Frank HR, Sequeira S, et al. A latent variable approach to differentiating neural mechanisms of irritability and anxiety in youth. JAMA Psychiatry. 2018;75:631–9. 10.1001/JAMAPSYCHIATRY.2018.0468. 29625429 10.1001/jamapsychiatry.2018.0468 PMC6137523

[69] Dougherty LR, Schwartz K, Kryza-Lacombe M, Weisberg J, Spechler PA, Wiggins JL. Preschool-and school-age irritability predict reward-related brain function. J Am Acad Child Adolesc Psychiatry. 2018;57:407–17. 10.1016/j.jaac.2018.03.012. 29859556 10.1016/j.jaac.2018.03.012

[70] Kashkouli Nejad K, Sugiura M, Nozawa T, Kotozaki Y, Furusawa Y, Nishino K, et al. Supramarginal activity in interoceptive attention tasks. Neurosci Lett. 2015;589:42–6. 10.1016/J.NEULET.2015.01.031. 25596444 10.1016/j.neulet.2015.01.031

[71] Leech R, Sharp DJ. The role of the posterior cingulate cortex in cognition and disease. Brain. 2014;137:12–32. 10.1093/brain/awt162. 23869106 10.1093/brain/awt162 PMC3891440

[72] Liuzzi MT, Kryza-Lacombe M, Christian IR, Owen C, Redcay E, Riggins T, et al. Irritability in early to middle childhood: cross-sectional and longitudinal associations with resting state amygdala and ventral striatum connectivity. Dev Cogn Neurosci. 2023;60:101206. 10.1016/j.dcn.2023.101206. 36736018 10.1016/j.dcn.2023.101206 PMC9918422

[73] Klein DN, Dougherty LR, Kessel EM, Silver J, Carlson GA. A transdiagnostic perspective on youth irritability. Curr Dir Psychol Sci. 2021;30:437–43. 10.1177/09637214211035101. 35046617 10.1177/09637214211035101 PMC8765598

[74] Finlay-Jones AL, Ang JE, Brook J, Lucas JD, MacNeill LA, Mancini VO, et al. Systematic review and meta-analysis: early irritability as a transdiagnostic neurodevelopmental vulnerability to later mental health problems. J Am Acad Child Adolesc Psychiatry. 2023;63:184–215. 10.1016/J.JAAC.2023.01.018. 36863413 10.1016/j.jaac.2023.01.018 PMC10460834

[75] Tseng WL, Deveney C, Brotman M, Stoddard J, Moroney E, Machlin L, et al. 37. Neural mechanisms of frustration and irritability across diagnoses. Biol Psychiatry. 2017;81:S16.

[76] Liu P, Tu H, Zhang A, Yang C, Liu Z, Lei L, et al. Brain functional alterations in MDD patients with somatic symptoms: a resting-state fMRI study. J Affect Disord. 2021;295:788–96. 10.1016/J.JAD.2021.08.143. 34517253 10.1016/j.jad.2021.08.143

[77] Salvadore G, Nugent AC, Lemaitre H, Luckenbaugh DA, Tinsley R, Cannon DM, et al. Prefrontal cortical abnormalities in currently depressed versus currently remitted patients with major depressive disorder. Neuroimage. 2011;54:2643–51. 10.1016/J.NEUROIMAGE.2010.11.011. 21073959 10.1016/j.neuroimage.2010.11.011 PMC3020249

[78] Lake AJ, Finn PR, James TW. Neural correlates of emotion reappraisal in individuals with externalizing psychopathology. Brain Imaging Behav. 2017;11:76–85. 10.1007/s11682-015-9500-7. 26809287 10.1007/s11682-015-9500-7 PMC4959999

[79] Judd LL, Schettler PJ, Akiskal H, Coryell W, Fawcett J, Fiedorowicz JG, et al. Prevalence and clinical significance of subsyndromal manic symptoms, including irritability and psychomotor agitation, during bipolar major depressive episodes. J Affect Disord. 2012;138:440–8. 10.1016/J.JAD.2011.12.046. 22314261 10.1016/j.jad.2011.12.046 PMC3677770

[80] Grossman RA, Ehrenreich-May J. Using the unified protocol for transdiagnostic treatment of emotional disorders with youth exhibiting anger and irritability. Cogn Behav Pract. 2020;27:184–201. 10.1016/J.CBPRA.2019.05.004.

[81] Singh MK, Hu R, Miklowitz DJ. Preventing irritability and temper outbursts in youth by building resilience. Child Adolesc Psychiatr Clin N Am. 2021;30:595–610. 10.1016/J.CHC.2021.04.009. 34053688 10.1016/j.chc.2021.04.009 PMC8184316

[82] Mallidi A, Meza-Cervera T, Kircanski K, Stringaris A, Brotman MA, Pine DS, et al. Robust caregiver-youth discrepancies in irritability ratings on the affective reactivity index: an investigation of its origins. J Affect Disord. 2023;332:185–93. 10.1016/J.JAD.2023.03.091. 37030330 10.1016/j.jad.2023.03.091 PMC10170868

[83] Barch DM, Albaugh MD, Baskin-Sommers A, Bryant BE, Clark DB, Dick AS, et al. Demographic and mental health assessments in the adolescent brain and cognitive development study: updates and age-related trajectories. Dev Cogn Neurosci. 2021;52:1–44. 10.1016/j.dcn.2021.101031. 10.1016/j.dcn.2021.101031 PMC8579129 34742018

[84] Cardinale EM, Freitag GF, Brotman MA, Pine DS, Leibenluft E, Kircanski K. Phasic versus tonic irritability: differential associations with attention-deficit/hyperactivity disorder symptoms. J Am Acad Child Adolesc Psychiatry. 2021;60:1513–23. 10.1016/J.JAAC.2020.11.022. 33440203 10.1016/j.jaac.2020.11.022 PMC9073575

[85] Carlson GA, Silver J, Klein DN. Psychometric properties of the emotional outburst inventory (EMO-I): rating what children do when they are irritable. J Clin Psychiatry. 2022;83:39382 10.4088/JCP.21M14015. 10.4088/JCP.21m14015 35081279

[86] Paulus MP, Thompson WK. The challenges and opportunities of small effects: the new normal in academic psychiatry. JAMA Psychiatry. 2019;76:353–54. 10.1001/jamapsychiatry.2018.4540. 30810720 10.1001/jamapsychiatry.2018.4540

