# Supplementary material for: Concurrent and longitudinal neurostructural correlates of irritability in children
Source: Neuropsychopharmacology. 2024 Aug 17;49(13):2069–76. doi: 10.1038/s41386-024-01966-4 (PMC11480493; doi:10.1038/s41386-024-01966-4)
Supplement: Supplementary file 1 — Supplementary Materials for Concurrent and Longitudinal Neurostructural Correlates of Irritability in Children [file 41386_2024_1966_MOESM1_ESM.docx]

**Supplementary Materials for**

**Concurrent and Longitudinal Neurostructural Correlates of Irritability in Children**

Table of Contents

[Generalizability of the ABCD sample 2](#_Toc173507143)

[Latent irritability factor 2](#_Toc173507144)

[Image acquisition, quality assurance, and processing 2](#_Toc173507145)

[Supplementary Table 1 5](#_Toc173507146)

[Associations between irritability and gray matter volume in 68 cortical regions and 19 subcortical regions 5](#_Toc173507147)

[Supplementary Table 2 8](#_Toc173507148)

[Associations between irritability and gray matter volume in 68 cortical regions and 19 subcortical regions with anxiety symptoms as a covariate 8](#_Toc173507149)

[Supplementary Table 3 11](#_Toc173507150)

[Associations between irritability and gray matter volume in 68 cortical regions and 19 subcortical regions with depression symptoms as a covariate 11](#_Toc173507151)

[Supplementary Table 4 14](#_Toc173507152)

[Associations between irritability and gray matter volume in 68 cortical regions and 19 subcortical regions with total psychopathology symptoms as a covariate 14](#_Toc173507153)

[Supplementary Table 5 17](#_Toc173507154)

[Gray matter volume in 68 cortical and 19 subcortical regions as predictors of irritability at the third-year follow-up 17](#_Toc173507155)

[Supplementary Table 6 20](#_Toc173507156)

[Associations between irritability and cortical thickness in 68 cortical regions 20](#_Toc173507157)

[Supplementary Table 7 22](#_Toc173507158)

[Associations between irritability and surface area in 68 cortical regions 22](#_Toc173507159)

[Supplementary Figure 1 24](#_Toc173507160)

# **Generalizability of the ABCD sample**

Procedures for the sampling and recruitment of participants for the ABCD Study is provided in greater detail in previous work (Garavan et al., 2018). In sum, across the 21 data collection sites, ABCD Study researchers conducted probability sampling of schools that were within each of the catchment areas. Children from each school that were eligible for the ABCD Study were recruited for study participation. Several sociodemographic variables were considered during recruitment such as age, sex, socioeconomic status, race/ethnicity, and urbanicity. Target numbers for these various demographic considerations were based on the American Community Survey (ACS) (an annual U.S. Census Bureau survey) and data on school enrollment from the National Center for Education Statistics. While the ABCD Study sites themselves are not representative of the general U.S. population, the recruitment process was consistent across each site and unbiased. In addition, the ABCD Study generated and provides post-stratification weights that can make the data more representative of the US population. These propensity weighting methods calibrate the ABCD Study sample based on nationally-representative data and can be used in analyses to attenuate potential demographic and socioeconomic selection biases (Heeringa & Berglund, 2020).

# **Latent irritability factor**

All five CBCL irritability items had sufficient endorsement across response options and were not redundant (polychoric correlations did not exceed .72). An item-factor analysis revealed that the items represent a unidimensional scale as shown by a clear elbow in the scree plot after one factor (Figure 1b). Additionally, only the first factor exceeded an eigenvalue of 1. Eigenvalues indicate the amount of variance explained by each factor and eigenvalues greater than 1 are typically retained. In addition, the ratio of the first to second eigenvalues showed a value of 7.36, which is greater than the traditional cutoff of 3 for determining whether more than one factor may be present in the data, further supporting that these items reflect a unidimensional scale. We then used a confirmatory factor analysis to derive a single latent variable from the five items that represents the degree of irritability (for standardized loadings, see Figure 1a). The latent factor score for each participant represents a weighted sum of these variables, with higher scores indicating greater irritability symptoms, while also accounting for measurement error. Model fit was assessed using the following criteria: comparative fit index (CFI) ≥ 0.9, root mean square error of approximation (RMSEA) ≤ .06, and standardized root mean square residual (SRMR) ≤ .08 (Jackson et al., 2009; McDonald & Ho, 2002). The confirmatory factor model showed adequate fit at baseline (CFI = .996; RMSEA = .04; SRMR = .02) and at the third-year follow-up (CFI = .991; RMSEA = .06; SRMR = .02).

# **Image acquisition, quality assurance, and processing**

The ABCD Data Analysis and Informatics Center (DAIC) and the ABCD Imaging Acquisition Workgroup developed an imaging protocol to standardize collection across multiple 3 tesla (3T) scanner platforms (Siemens Prisma, General Electric (GE) 750, and Phillips) across 21 data collection sites (Casey et al., 2018). 3D T1- and T2-weighted images of brain structure were collected. Whole brain T1-weighted images were acquired using the following parameters: TR (repetition time) 2400 to 2500 ms; TE (echo time) 2 to 2.9 ms; FOV (field of view) 256 × 240 to 256; FOV phase of 93.75% to 100%; matrix 256 × 256; 176 to 225 slices; TI (inversion delay) 1060 ms; flip angle of 8°; voxel resolution of 1×1×1×mm; total acquisition time was 7 minutes and 12 seconds for Siemens Prisma, 6 minutes and 9 seconds for GE 750, and 5 minutes and 38 seconds for Phillips.

For processing and analysis of imaging data, DAIC used the Multi-Modal Processing Stream (MMPS), a software package developed and continued at the Center for Multimodal Imaging and Genetic (CMIG) at the University of California, San Diego (UCSD) for centralized processing and analysis. Data underwent preprocessing which included correction for gradient nonlinearity distortions, intensity scaling and homogeneity correction, registration to an averaged reference brain in standard space, and manual quality control (QC). Cortical surface reconstruction and subcortical segmentation were also completed using automated, atlas-based, segmentation procedures in FreeSurfer v.5.3. Next, the morphometric measures were calculated with average cortical thickness and average volume in each cortical parcel of the standard FreeSurfer Desikan-Killiany parcellation scheme (Desikan et al., 2006) and the average volume in each subcortical region (Fischl et al., 2002). Post-processing QC was then completed by trained technicians for motion, intensity homogeneity, white matter underestimation, pial overestimation, and magnetic susceptibility artifact. All processing of imaging data was executed by DAIC.

**References**

Casey, B. J., Cannonier, T., Conley, M. I., Cohen, A. O., Barch, D. M., Heitzeg, M. M., Soules, M. E., Teslovich, T., Dellarco, D. V., Garavan, H., Orr, C. A., Wager, T. D., Banich, M. T., Speer, N. K., Sutherland, M. T., Riedel, M. C., Dick, A. S., Bjork, J. M., Thomas, K. M., … Dale, A. M. (2018). The Adolescent Brain Cognitive Development (ABCD) study: Imaging acquisition across 21 sites. *Developmental Cognitive Neuroscience*, *32*, 43–54. https://doi.org/10.1016/j.dcn.2018.03.001

Desikan, R. S., Ségonne, F., Fischl, B., Quinn, B. T., Dickerson, B. C., Blacker, D., Buckner, R. L., Dale, A. M., Maguire, R. P., Hyman, B. T., Albert, M. S., & Killiany, R. J. (2006). An automated labeling system for subdividing the human cerebral cortex on MRI scans into gyral based regions of interest. *NeuroImage*, *31*(3), 968–980. https://doi.org/10.1016/j.neuroimage.2006.01.021

Fischl, B., Salat, D., Busa, E., Albert, M., Dieterich, M., & Haselgrove, C. (2002). Whole Brain Segmentation: Automated Labeling of Neuroanatomical Structures in the Human Brain. *Neuron*, *33*(3), 341–355. https://doi.org/10.1016/S0896-6273(02)00569-X

Garavan, H., Bartsch, H., Conway, K., Decastro, A., Goldstein, R. Z., Heeringa, S., Jernigan, T., Potter, A., Thompson, W., & Zahs, D. (2018). Recruiting the ABCD sample: Design considerations and procedures. *Developmental Cognitive Neuroscience*, *32*, 16–22. https://doi.org/10.1016/J.DCN.2018.04.004

Heeringa, S. G., & Berglund, P. A. (2020). A Guide for Population-based Analysis of the Adolescent Brain Cognitive Development (ABCD) Study Baseline Data. *BioRxiv*, 2020–02. https://doi.org/10.1101/2020.02.10.942011

Jackson, D. L., Gillaspy, J. A., & Purc-Stephenson, R. (2009). Reporting Practices in Confirmatory Factor Analysis: An Overview and Some Recommendations. *Psychological Methods*, *14*(1). https://doi.org/10.1037/a0014694

McDonald, R. P., & Ho, M. H. R. (2002). Principles and practice in reporting structural equation analyses. *Psychological Methods*, *7*(1). https://doi.org/10.1037/1082-989X.7.1.64

# **Supplementary Table 1**

## *Associations between irritability and gray matter volume in 68 cortical regions and 19 subcortical regions*

|  | *β* | *SE* | *z* | *p* | *p_fdr_* | *R^2^* |
| --- | --- | --- | --- | --- | --- | --- |
| Left banks of superior temporal sulcus | -0.039 | 0.02 | -2.64 | .008 | .056 | .002 |
| Left caudal anterior cingulate | -0.016 | 0.02 | -1.11 | .268 | .432 | .000 |
| Left caudal middle frontal | 0.004 | 0.02 | 0.26 | .796 | .888 | .000 |
| Left cuneus | 0.020 | 0.02 | 1.38 | .167 | .323 | .000 |
| Left entorhinal | -0.020 | 0.01 | -1.44 | .151 | .312 | .000 |
| Left fusiform | -0.041 | 0.02 | -2.36 | .018 | .097 | .002 |
| **Left inferior parietal** | **-0.052** | **0.02** | **-3.23** | **.001** | **.010** | **.003** |
| Left inferior temporal | -0.041 | 0.02 | -2.34 | .019 | .097 | .002 |
| Left isthmus cingulate | -0.022 | 0.02 | -1.36 | .172 | .324 | .000 |
| Left lateral occipital | -0.013 | 0.02 | -0.80 | .423 | .614 | .000 |
| Left lateral orbitofrontal | -0.014 | 0.02 | -0.74 | .460 | .614 | .000 |
| Left lingual | 0.009 | 0.02 | 0.62 | .538 | .669 | .000 |
| Left medial orbitofrontal | -0.002 | 0.02 | -0.12 | .903 | .947 | .000 |
| Left middle temporal | -0.039 | 0.02 | -2.24 | .025 | .109 | .002 |
| Left parahippocampal | 0.000 | 0.02 | 0.02 | .983 | .992 | .000 |
| Left paracentral | -0.018 | 0.02 | -1.14 | .255 | .427 | .000 |
| Left pars opercularis | 0.004 | 0.02 | 0.29 | .771 | .871 | .000 |
| Left pars orbitalis | -0.019 | 0.02 | -1.19 | .233 | .397 | .000 |
| Left pars triangularis | 0.011 | 0.02 | 0.73 | .466 | .614 | .000 |
| Left pericalcarine | 0.021 | 0.01 | 1.50 | .133 | .289 | .000 |
| **Left postcentral** | **-0.048** | **0.02** | **-2.79** | **.005** | **.040** | **.002** |
| Left posterior cingulate | -0.027 | 0.02 | -1.66 | .098 | .258 | .001 |
| **Left precentral** | **-0.059** | **0.02** | **-3.40** | **.001** | **.010** | **.003** |
| Left precuneus | -0.024 | 0.02 | -1.32 | .186 | .330 | .001 |
| Left rostral anterior cingulate | -0.025 | 0.02 | -1.53 | .126 | .288 | .001 |
| Left rostral middle frontal | -0.034 | 0.02 | -2.00 | .046 | .154 | .001 |
| Left superior frontal | -0.037 | 0.02 | -1.95 | .051 | .164 | .001 |
| Left superior parietal | -0.011 | 0.02 | -0.66 | .511 | .654 | .000 |
| **Left superior temporal** | **-0.066** | **0.02** | **-3.79** | **.000** | **.000** | **.004** |
| **Left supramarginal** | **-0.062** | **0.02** | **-3.89** | **.000** | **.000** | **.004** |
| Left frontal pole | 0.007 | 0.02 | 0.49 | .621 | .744 | .000 |
| Left temporal pole | -0.032 | 0.02 | -2.14 | .032 | .121 | .001 |
| Left transverse temporal | -0.020 | 0.02 | -1.34 | .179 | .324 | .000 |
| Left insula | -0.040 | 0.02 | -2.25 | .024 | .109 | .002 |
| Right banks of superior temporal sulcus | -0.031 | 0.02 | -2.02 | .044 | .154 | .001 |
| Right caudal anterior cingulate | -0.020 | 0.02 | -1.35 | .177 | .324 | .000 |
| Right caudal middle frontal | -0.026 | 0.02 | -1.67 | .095 | .258 | .001 |
| Right cuneus | 0.013 | 0.02 | 0.86 | .389 | .594 | .000 |
| Right entorhinal | -0.021 | 0.01 | -1.43 | .154 | .312 | .000 |
| Right fusiform | -0.037 | 0.02 | -1.99 | .046 | .154 | .001 |
| **Right inferior parietal** | **-0.063** | **0.02** | **-3.78** | **.000** | **.000** | **.004** |
| **Right inferior temporal** | **-0.058** | **0.02** | **-3.31** | **.001** | **.010** | **.003** |
| Right isthmus cingulate | -0.001 | 0.02 | -0.07 | .941 | .975 | .000 |
| Right lateral occipital | -0.004 | 0.02 | -0.22 | .827 | .899 | .000 |
| Right lateral orbitofrontal | -0.030 | 0.02 | -1.54 | .124 | .288 | .001 |
| Right lingual | 0.012 | 0.02 | 0.78 | .436 | .614 | .000 |
| Right medial orbitofrontal | -0.029 | 0.02 | -1.66 | .096 | .258 | .001 |
| **Right middle temporal** | **-0.075** | **0.02** | **-4.09** | **.000** | **.000** | **.006** |
| Right parahippocampal | -0.011 | 0.02 | -0.74 | .462 | .614 | .000 |
| Right paracentral | -0.025 | 0.02 | -1.60 | .110 | .273 | .001 |
| Right pars opercularis | 0.005 | 0.02 | 0.34 | .735 | .841 | .000 |
| Right pars orbitalis | 0.001 | 0.02 | 0.06 | .953 | .975 | .000 |
| Right pars triangularis | 0.011 | 0.02 | 0.75 | .455 | .614 | .000 |
| Right pericalcarine | 0.018 | 0.01 | 1.22 | .221 | .385 | .000 |
| **Right postcentral** | **-0.053** | **0.02** | **-3.07** | **.002** | **.017** | **.003** |
| **Right posterior cingulate** | **-0.057** | **0.02** | **-3.47** | **.001** | **.010** | **.003** |
| **Right precentral** | **-0.073** | **0.02** | **-4.27** | **.000** | **.000** | **.005** |
| Right precuneus | -0.003 | 0.02 | -0.16 | .873 | .926 | .000 |
| Right rostral anterior cingulate | -0.003 | 0.02 | -0.22 | .823 | .899 | .000 |
| Right rostral middle frontal | -0.012 | 0.02 | -0.74 | .460 | .614 | .000 |
| Right superior frontal | -0.039 | 0.02 | -2.15 | .031 | .121 | .002 |
| Right superior parietal | 0.013 | 0.02 | 0.78 | .436 | .614 | .000 |
| Right superior temporal | -0.047 | 0.02 | -2.62 | .009 | .056 | .002 |
| Right supramarginal | -0.041 | 0.02 | -2.61 | .009 | .056 | .002 |
| Right frontal pole | -0.024 | 0.02 | -1.61 | .107 | .273 | .001 |
| Right temporal pole | 0.009 | 0.01 | 0.61 | .545 | .669 | .000 |
| Right transverse temporal | -0.022 | 0.02 | -1.39 | .163 | .322 | .000 |
| Right insula | -0.033 | 0.02 | -1.80 | .072 | .224 | .001 |
| Left cerebellum cortex | -0.030 | 0.02 | -1.50 | .133 | .289 | .001 |
| Left thalamus proper | -0.047 | 0.02 | -2.37 | .018 | .097 | .002 |
| Left caudate | -0.026 | 0.02 | -1.55 | .121 | .288 | .001 |
| Left putamen | -0.010 | 0.02 | -0.60 | .546 | .669 | .000 |
| Left pallidum | 0.003 | 0.02 | 0.16 | .872 | .926 | .000 |
| Brain stem | -0.018 | 0.02 | -0.89 | .372 | .578 | .000 |
| Left hippocampus | -0.039 | 0.02 | -2.15 | .032 | .121 | .002 |
| Left amygdala | 0.000 | 0.02 | -0.01 | .992 | .992 | .000 |
| Left accumbens area | -0.024 | 0.02 | -1.45 | .146 | .310 | .001 |
| Left ventral diencephalon | -0.036 | 0.02 | -1.70 | .090 | .258 | .001 |
| Right cerebellum cortex | -0.022 | 0.02 | -1.09 | .276 | .437 | .000 |
| Right thalamus proper | -0.022 | 0.02 | -1.11 | .268 | .432 | .000 |
| Right caudate | -0.006 | 0.02 | -0.37 | .715 | .829 | .000 |
| Right putamen | -0.014 | 0.02 | -0.81 | .418 | .614 | .000 |
| Right pallidum | -0.007 | 0.02 | -0.41 | .682 | .802 | .000 |
| Right hippocampus | -0.029 | 0.02 | -1.66 | .098 | .258 | .001 |
| Right amygdala | -0.009 | 0.02 | -0.49 | .624 | .744 | .000 |
| Right accumbens area | -0.037 | 0.02 | -2.28 | .022 | .106 | .001 |
| Right ventral diencephalon | -0.015 | 0.02 | -0.71 | .477 | .619 | .000 |

# **Supplementary Table 2**

## *Associations between irritability and gray matter volume in 68 cortical regions and 19 subcortical regions with anxiety symptoms as a covariate*

|  | *β* | *SE* | *z* | *p* | *p_fdr_* | *R^2^* |
| --- | --- | --- | --- | --- | --- | --- |
| Left banks of superior temporal sulcus | -0.033 | 0.01 | -2.53 | .011 | .072 | .001 |
| Left caudal anterior cingulate | -0.016 | 0.01 | -1.23 | .220 | .425 | .000 |
| Left caudal middle frontal | -0.010 | 0.01 | -0.72 | .474 | .649 | .000 |
| Left cuneus | 0.008 | 0.01 | 0.59 | .553 | .725 | .000 |
| Left entorhinal | -0.010 | 0.01 | -0.80 | .421 | .630 | .000 |
| **Left fusiform** | **-0.044** | **0.02** | **-2.86** | **.004** | **.000** | **.002** |
| **Left inferior parietal** | **-0.046** | **0.01** | **-3.34** | **.001** | **.000** | **.002** |
| Left inferior temporal | -0.030 | 0.02 | -1.94 | .052 | .161 | .001 |
| Left isthmus cingulate | -0.023 | 0.01 | -1.60 | .109 | .259 | .001 |
| Left lateral occipital | -0.026 | 0.01 | -1.77 | .077 | .205 | .001 |
| Left lateral orbitofrontal | -0.023 | 0.02 | -1.36 | .175 | .382 | .001 |
| Left lingual | 0.000 | 0.01 | 0.00 | .997 | 1.000 | .000 |
| Left medial orbitofrontal | 0.014 | 0.02 | 0.86 | .392 | .621 | .000 |
| Left middle temporal | -0.034 | 0.02 | -2.24 | .025 | .137 | .001 |
| Left parahippocampal | 0.005 | 0.01 | 0.38 | .701 | .781 | .000 |
| Left paracentral | -0.011 | 0.01 | -0.85 | .397 | .621 | .000 |
| Left pars opercularis | 0.005 | 0.01 | 0.42 | .673 | .757 | .000 |
| Left pars orbitalis | -0.014 | 0.01 | -1.05 | .296 | .529 | .000 |
| Left pars triangularis | 0.007 | 0.01 | 0.58 | .560 | .727 | .000 |
| Left pericalcarine | 0.000 | 0.01 | -0.02 | .986 | 1.000 | .000 |
| Left postcentral | -0.019 | 0.02 | -1.25 | .211 | .415 | .000 |
| Left posterior cingulate | -0.024 | 0.01 | -1.71 | .087 | .217 | .001 |
| **Left precentral** | **-0.048** | **0.02** | **-3.15** | **.002** | **.000** | **.002** |
| Left precuneus | -0.028 | 0.02 | -1.83 | .067 | .196 | .001 |
| Left rostral anterior cingulate | -0.024 | 0.01 | -1.72 | .085 | .217 | .001 |
| Left rostral middle frontal | -0.026 | 0.02 | -1.77 | .077 | .205 | .001 |
| Left superior frontal | -0.033 | 0.02 | -2.06 | .040 | .158 | .001 |
| Left superior parietal | -0.011 | 0.01 | -0.76 | .449 | .645 | .000 |
| **Left superior temporal** | **-0.050** | **0.02** | **-3.25** | **.001** | **.000** | **.003** |
| **Left supramarginal** | **-0.044** | **0.01** | **-3.11** | **.002** | **.000** | **.002** |
| Left frontal pole | 0.006 | 0.01 | 0.45 | .655 | .757 | .000 |
| Left temporal pole | -0.027 | 0.01 | -2.05 | .040 | .158 | .001 |
| Left transverse temporal | -0.026 | 0.01 | -1.97 | .049 | .161 | .001 |
| Left insula | -0.038 | 0.02 | -2.41 | .016 | .109 | .001 |
| **Right banks of superior temporal sulcus** | **-0.042** | **0.01** | **-3.16** | **.002** | **.000** | **.002** |
| Right caudal anterior cingulate | -0.007 | 0.01 | -0.54 | .591 | .744 | .000 |
| Right caudal middle frontal | -0.026 | 0.01 | -1.92 | .055 | .174 | .001 |
| Right cuneus | -0.002 | 0.01 | -0.16 | .873 | .923 | .000 |
| Right entorhinal | -0.005 | 0.01 | -0.43 | .668 | .757 | .000 |
| Right fusiform | -0.031 | 0.02 | -1.96 | .050 | .161 | .001 |
| **Right inferior parietal** | **-0.066** | **0.01** | **-4.62** | **.000** | **.000** | **.004** |
| Right inferior temporal | -0.028 | 0.02 | -1.85 | .064 | .174 | .001 |
| Right isthmus cingulate | -0.011 | 0.01 | -0.81 | .417 | .630 | .000 |
| Right lateral occipital | -0.016 | 0.02 | -1.03 | .304 | .529 | .000 |
| Right lateral orbitofrontal | -0.031 | 0.02 | -1.87 | .062 | .174 | .001 |
| Right lingual | 0.010 | 0.01 | 0.78 | .439 | .645 | .000 |
| Right medial orbitofrontal | -0.024 | 0.02 | -1.55 | .122 | .275 | .001 |
| **Right middle temporal** | **-0.062** | **0.02** | **-3.88** | **.000** | **.000** | **.004** |
| Right parahippocampal | -0.013 | 0.01 | -1.01 | .314 | .529 | .000 |
| Right paracentral | -0.012 | 0.01 | -0.88 | .382 | .621 | .000 |
| Right pars opercularis | 0.006 | 0.01 | 0.50 | .619 | .757 | .000 |
| Right pars orbitalis | 0.003 | 0.01 | 0.24 | .811 | .870 | .000 |
| Right pars triangularis | 0.014 | 0.01 | 1.10 | .273 | .500 | .000 |
| Right pericalcarine | -0.001 | 0.01 | -0.09 | .926 | .952 | .000 |
| Right postcentral | -0.033 | 0.02 | -2.21 | .027 | .137 | .001 |
| **Right posterior cingulate** | **-0.047** | **0.01** | **-3.27** | **.001** | **.000** | **.002** |
| **Right precentral** | **-0.060** | **0.02** | **-4.01** | **.000** | **.000** | **.004** |
| Right precuneus | -0.010 | 0.02 | -0.65 | .516 | .707 | .000 |
| Right rostral anterior cingulate | -0.007 | 0.01 | -0.53 | .595 | .746 | .000 |
| Right rostral middle frontal | -0.005 | 0.01 | -0.37 | .715 | .783 | .000 |
| Right superior frontal | -0.037 | 0.02 | -2.38 | .018 | .109 | .001 |
| Right superior parietal | 0.010 | 0.01 | 0.74 | .459 | .645 | .000 |
| Right superior temporal | -0.044 | 0.02 | -2.78 | .005 | .072 | .002 |
| Right supramarginal | -0.033 | 0.01 | -2.33 | .020 | .109 | .001 |
| Right frontal pole | -0.012 | 0.01 | -0.94 | .349 | .586 | .000 |
| Right temporal pole | 0.009 | 0.01 | 0.74 | .460 | .645 | .000 |
| Right transverse temporal | -0.019 | 0.01 | -1.36 | .174 | .370 | .000 |
| Right insula | -0.016 | 0.02 | -1.02 | .306 | .529 | .000 |
| Left cerebellum cortex | -0.040 | 0.02 | -2.35 | .019 | .109 | .002 |
| Left thalamus proper | -0.037 | 0.02 | -2.17 | .030 | .137 | .001 |
| Left caudate | -0.009 | 0.01 | -0.63 | .530 | .709 | .000 |
| Left putamen | 0.008 | 0.01 | 0.54 | .587 | .744 | .000 |
| Left pallidum | 0.002 | 0.01 | 0.12 | .901 | .943 | .000 |
| Brain stem | -0.025 | 0.02 | -1.45 | .148 | .335 | .001 |
| Left hippocampus | -0.032 | 0.02 | -2.01 | .045 | .161 | .001 |
| Left amygdala | 0.007 | 0.02 | 0.46 | .645 | .757 | .000 |
| Left accumbens area | -0.018 | 0.01 | -1.25 | .211 | .415 | .000 |
| Left ventral diencephalon | -0.032 | 0.02 | -1.73 | .084 | .205 | .001 |
| Right cerebellum cortex | -0.036 | 0.02 | -2.11 | .035 | .158 | .001 |
| Right thalamus proper | -0.015 | 0.02 | -0.84 | .400 | .621 | .000 |
| Right caudate | 0.007 | 0.01 | 0.48 | .633 | .757 | .000 |
| Right putamen | 0.007 | 0.02 | 0.46 | .643 | .757 | .000 |
| Right pallidum | 0.001 | 0.01 | 0.09 | .925 | .952 | .000 |
| Right hippocampus | -0.020 | 0.02 | -1.29 | .198 | .414 | .000 |
| Right amygdala | -0.005 | 0.02 | -0.36 | .717 | .783 | .000 |
| Right accumbens area | -0.028 | 0.01 | -1.94 | .052 | .161 | .001 |
| Right ventral diencephalon | -0.020 | 0.02 | -1.11 | .266 | .500 | .000 |

# **Supplementary Table 3**

## *Associations between irritability and gray matter volume in 68 cortical regions and 19 subcortical regions with depression symptoms as a covariate*

|  | *β* | *SE* | *z* | *p* | *p_fdr_* | *R^2^* |
| --- | --- | --- | --- | --- | --- | --- |
| Left banks of superior temporal sulcus | -0.033 | 0.01 | -2.59 | .010 | .087 | .001 |
| Left caudal anterior cingulate | -0.013 | 0.01 | -1.06 | .287 | .587 | .000 |
| Left caudal middle frontal | -0.006 | 0.01 | -0.47 | .640 | .884 | .000 |
| Left cuneus | 0.018 | 0.01 | 1.45 | .148 | .386 | .000 |
| Left entorhinal | -0.030 | 0.01 | -2.51 | .012 | .087 | .001 |
| Left fusiform | -0.037 | 0.02 | -2.47 | .014 | .094 | .001 |
| Left inferior parietal | -0.038 | 0.01 | -2.80 | .005 | .072 | .001 |
| Left inferior temporal | -0.027 | 0.02 | -1.79 | .074 | .280 | .001 |
| Left isthmus cingulate | -0.017 | 0.01 | -1.21 | .226 | .504 | .000 |
| Left lateral occipital | -0.015 | 0.01 | -1.06 | .289 | .587 | .000 |
| Left lateral orbitofrontal | -0.017 | 0.02 | -1.04 | .300 | .593 | .000 |
| Left lingual | 0.008 | 0.01 | 0.59 | .556 | .849 | .000 |
| Left medial orbitofrontal | 0.006 | 0.02 | 0.38 | .706 | .889 | .000 |
| Left middle temporal | -0.021 | 0.02 | -1.39 | .165 | .410 | .000 |
| Left parahippocampal | 0.005 | 0.01 | 0.41 | .679 | .889 | .000 |
| Left paracentral | -0.003 | 0.01 | -0.20 | .839 | .916 | .000 |
| Left pars opercularis | 0.000 | 0.01 | -0.01 | .990 | .995 | .000 |
| Left pars orbitalis | -0.022 | 0.01 | -1.68 | .093 | .289 | .000 |
| Left pars triangularis | 0.000 | 0.01 | 0.03 | .976 | .995 | .000 |
| Left pericalcarine | 0.015 | 0.01 | 1.27 | .206 | .484 | .000 |
| Left postcentral | -0.005 | 0.02 | -0.34 | .736 | .889 | .000 |
| Left posterior cingulate | -0.020 | 0.01 | -1.44 | .151 | .386 | .000 |
| Left precentral | -0.038 | 0.02 | -2.51 | .012 | .087 | .001 |
| Left precuneus | -0.014 | 0.02 | -0.95 | .342 | .633 | .000 |
| Left rostral anterior cingulate | -0.026 | 0.01 | -1.91 | .057 | .225 | .001 |
| Left rostral middle frontal | -0.032 | 0.01 | -2.19 | .029 | .148 | .001 |
| Left superior frontal | -0.032 | 0.02 | -2.02 | .043 | .197 | .001 |
| Left superior parietal | -0.003 | 0.01 | -0.20 | .845 | .916 | .000 |
| **Left superior temporal** | **-0.056** | **0.02** | **-3.69** | **.000** | **.000** | **.003** |
| Left supramarginal | -0.038 | 0.01 | -2.74 | .006 | .075 | .001 |
| Left frontal pole | -0.007 | 0.01 | -0.53 | .593 | .860 | .000 |
| Left temporal pole | -0.028 | 0.01 | -2.22 | .027 | .147 | .001 |
| Left transverse temporal | -0.013 | 0.01 | -1.00 | .316 | .611 | .000 |
| Left insula | -0.037 | 0.02 | -2.38 | .017 | .106 | .001 |
| Right banks of superior temporal sulcus | -0.035 | 0.01 | -2.66 | .008 | .087 | .001 |
| Right caudal anterior cingulate | -0.003 | 0.01 | -0.26 | .797 | .889 | .000 |
| Right caudal middle frontal | -0.021 | 0.01 | -1.57 | .117 | .339 | .000 |
| Right cuneus | 0.007 | 0.01 | 0.55 | .580 | .855 | .000 |
| Right entorhinal | -0.009 | 0.01 | -0.71 | .479 | .772 | .000 |
| Right fusiform | -0.023 | 0.02 | -1.49 | .135 | .370 | .001 |
| **Right inferior parietal** | **-0.067** | **0.01** | **-4.65** | **.000** | **.000** | **.004** |
| Right inferior temporal | -0.035 | 0.02 | -2.34 | .019 | .110 | .001 |
| Right isthmus cingulate | -0.003 | 0.01 | -0.19 | .853 | .916 | .000 |
| Right lateral occipital | -0.011 | 0.02 | -0.71 | .478 | .772 | .000 |
| Right lateral orbitofrontal | -0.032 | 0.02 | -1.94 | .052 | .215 | .001 |
| Right lingual | 0.010 | 0.01 | 0.80 | .427 | .714 | .000 |
| Right medial orbitofrontal | -0.030 | 0.02 | -1.98 | .048 | .209 | .001 |
| **Right middle temporal** | **-0.056** | **0.02** | **-3.54** | **.000** | **.000** | **.003** |
| Right parahippocampal | -0.001 | 0.01 | -0.05 | .957 | .995 | .000 |
| Right paracentral | -0.006 | 0.01 | -0.42 | .675 | .889 | .000 |
| Right pars opercularis | 0.004 | 0.01 | 0.30 | .764 | .889 | .000 |
| Right pars orbitalis | -0.005 | 0.01 | -0.35 | .726 | .889 | .000 |
| Right pars triangularis | 0.013 | 0.01 | 1.06 | .290 | .587 | .000 |
| Right pericalcarine | 0.011 | 0.01 | 0.90 | .368 | .667 | .000 |
| Right postcentral | -0.025 | 0.02 | -1.71 | .087 | .284 | .001 |
| Right posterior cingulate | -0.042 | 0.01 | -3.00 | .003 | .052 | .002 |
| **Right precentral** | **-0.047** | **0.02** | **-3.19** | **.001** | **.022** | **.002** |
| Right precuneus | 0.008 | 0.02 | 0.48 | .631 | .884 | .000 |
| Right rostral anterior cingulate | -0.004 | 0.01 | -0.34 | .732 | .889 | .000 |
| Right rostral middle frontal | -0.018 | 0.01 | -1.29 | .198 | .478 | .000 |
| Right superior frontal | -0.032 | 0.02 | -2.06 | .039 | .188 | .001 |
| Right superior parietal | 0.024 | 0.01 | 1.72 | .086 | .284 | .001 |
| Right superior temporal | -0.039 | 0.02 | -2.53 | .011 | .087 | .002 |
| Right supramarginal | -0.021 | 0.01 | -1.49 | .136 | .370 | .000 |
| Right frontal pole | -0.012 | 0.01 | -0.95 | .340 | .633 | .000 |
| Right temporal pole | 0.013 | 0.01 | 1.08 | .282 | .587 | .000 |
| Right transverse temporal | -0.011 | 0.01 | -0.79 | .427 | .714 | .000 |
| Right insula | -0.010 | 0.02 | -0.64 | .525 | .830 | .000 |
| Left cerebellum cortex | -0.006 | 0.02 | -0.33 | .739 | .889 | .000 |
| Left thalamus proper | -0.029 | 0.02 | -1.72 | .086 | .284 | .001 |
| Left caudate | -0.001 | 0.01 | -0.08 | .939 | .995 | .000 |
| Left putamen | 0.000 | 0.01 | -0.01 | .995 | .995 | .000 |
| Left pallidum | 0.011 | 0.01 | 0.79 | .427 | .714 | .000 |
| Brain stem | 0.005 | 0.02 | 0.30 | .764 | .889 | .000 |
| Left hippocampus | -0.027 | 0.02 | -1.71 | .088 | .284 | .001 |
| Left amygdala | 0.006 | 0.02 | 0.36 | .718 | .889 | .000 |
| Left accumbens area | -0.001 | 0.01 | -0.05 | .961 | .995 | .000 |
| Left ventral diencephalon | -0.010 | 0.02 | -0.56 | .579 | .855 | .000 |
| Right cerebellum cortex | -0.004 | 0.02 | -0.26 | .795 | .889 | .000 |
| Right thalamus proper | 0.005 | 0.02 | 0.29 | .770 | .889 | .000 |
| Right caudate | 0.008 | 0.01 | 0.60 | .550 | .849 | .000 |
| Right putamen | 0.004 | 0.01 | 0.26 | .795 | .889 | .000 |
| Right pallidum | -0.007 | 0.01 | -0.52 | .605 | .863 | .000 |
| Right hippocampus | -0.019 | 0.02 | -1.24 | .213 | .488 | .000 |
| Right amygdala | -0.012 | 0.02 | -0.80 | .423 | .714 | .000 |
| Right accumbens area | -0.023 | 0.01 | -1.65 | .100 | .300 | .001 |
| Right ventral diencephalon | -0.007 | 0.02 | -0.37 | .713 | .889 | .000 |

# **Supplementary Table 4**

## *Associations between irritability and gray matter volume in 68 cortical regions and 19 subcortical regions with total psychopathology symptoms as a covariate*

|  | *β* | *SE* | *z* | *p* | *p_fdr_* | *R^2^* |
| --- | --- | --- | --- | --- | --- | --- |
| Left banks of superior temporal sulcus | -0.027 | 0.01 | -2.47 | .014 | .203 | .001 |
| Left caudal anterior cingulate | -0.005 | 0.01 | -0.47 | .638 | .894 | .000 |
| Left caudal middle frontal | -0.009 | 0.01 | -0.84 | .404 | .748 | .000 |
| Left cuneus | 0.013 | 0.01 | 1.17 | .244 | .685 | .000 |
| Left entorhinal | -0.012 | 0.01 | -1.18 | .239 | .685 | .000 |
| Left fusiform | -0.008 | 0.01 | -0.58 | .562 | .829 | .000 |
| Left inferior parietal | -0.027 | 0.01 | -2.32 | .021 | .261 | .001 |
| Left inferior temporal | -0.023 | 0.01 | -1.75 | .080 | .464 | .001 |
| Left isthmus cingulate | -0.011 | 0.01 | -0.93 | .353 | .731 | .000 |
| Left lateral occipital | -0.008 | 0.01 | -0.64 | .520 | .829 | .000 |
| Left lateral orbitofrontal | -0.005 | 0.01 | -0.37 | .709 | .929 | .000 |
| Left lingual | 0.016 | 0.01 | 1.41 | .157 | .613 | .000 |
| Left medial orbitofrontal | 0.003 | 0.01 | 0.19 | .849 | .929 | .000 |
| Left middle temporal | -0.018 | 0.01 | -1.41 | .159 | .613 | .000 |
| Left parahippocampal | 0.019 | 0.01 | 1.79 | .074 | .464 | .000 |
| Left paracentral | 0.002 | 0.01 | 0.19 | .847 | .929 | .000 |
| Left pars opercularis | -0.002 | 0.01 | -0.17 | .864 | .929 | .000 |
| Left pars orbitalis | -0.011 | 0.01 | -1.01 | .314 | .731 | .000 |
| Left pars triangularis | 0.005 | 0.01 | 0.44 | .658 | .894 | .000 |
| Left pericalcarine | 0.007 | 0.01 | 0.63 | .532 | .829 | .000 |
| Left postcentral | -0.001 | 0.01 | -0.11 | .915 | .937 | .000 |
| Left posterior cingulate | -0.006 | 0.01 | -0.53 | .597 | .866 | .000 |
| Left precentral | -0.020 | 0.01 | -1.54 | .123 | .594 | .000 |
| Left precuneus | -0.009 | 0.01 | -0.72 | .474 | .809 | .000 |
| Left rostral anterior cingulate | -0.013 | 0.01 | -1.10 | .272 | .696 | .000 |
| Left rostral middle frontal | -0.012 | 0.01 | -0.96 | .337 | .731 | .000 |
| Left superior frontal | -0.013 | 0.01 | -0.95 | .342 | .731 | .000 |
| Left superior parietal | -0.002 | 0.01 | -0.19 | .848 | .929 | .000 |
| Left superior temporal | -0.027 | 0.01 | -2.04 | .042 | .406 | .001 |
| Left supramarginal | -0.015 | 0.01 | -1.26 | .206 | .664 | .000 |
| Left frontal pole | -0.002 | 0.01 | -0.18 | .856 | .929 | .000 |
| Left temporal pole | -0.020 | 0.01 | -1.85 | .065 | .464 | .000 |
| Left transverse temporal | -0.011 | 0.01 | -0.98 | .329 | .731 | .000 |
| Left insula | -0.013 | 0.01 | -1.02 | .306 | .731 | .000 |
| Right banks of superior temporal sulcus | -0.029 | 0.01 | -2.59 | .010 | .203 | .001 |
| Right caudal anterior cingulate | 0.003 | 0.01 | 0.31 | .758 | .929 | .000 |
| Right caudal middle frontal | -0.019 | 0.01 | -1.72 | .086 | .468 | .000 |
| Right cuneus | 0.002 | 0.01 | 0.16 | .870 | .929 | .000 |
| Right entorhinal | 0.006 | 0.01 | 0.59 | .555 | .829 | .000 |
| Right fusiform | -0.004 | 0.01 | -0.29 | .774 | .929 | .000 |
| **Right inferior parietal** | **-0.048** | **0.01** | **-3.82** | **.000** | **.000** | **.002** |
| Right inferior temporal | -0.013 | 0.01 | -1.02 | .308 | .731 | .000 |
| Right isthmus cingulate | -0.010 | 0.01 | -0.84 | .403 | .748 | .000 |
| Right lateral occipital | -0.002 | 0.01 | -0.14 | .886 | .929 | .000 |
| Right lateral orbitofrontal | -0.019 | 0.01 | -1.33 | .184 | .632 | .000 |
| Right lingual | 0.016 | 0.01 | 1.39 | .165 | .613 | .000 |
| Right medial orbitofrontal | -0.023 | 0.01 | -1.77 | .077 | .464 | .001 |
| Right middle temporal | -0.035 | 0.01 | -2.55 | .011 | .203 | .001 |
| Right parahippocampal | 0.005 | 0.01 | 0.45 | .651 | .894 | .000 |
| Right paracentral | 0.001 | 0.01 | 0.12 | .907 | .937 | .000 |
| Right pars opercularis | -0.001 | 0.01 | -0.05 | .959 | .959 | .000 |
| Right pars orbitalis | 0.014 | 0.01 | 1.23 | .217 | .674 | .000 |
| Right pars triangularis | 0.013 | 0.01 | 1.19 | .234 | .685 | .000 |
| Right pericalcarine | 0.004 | 0.01 | 0.35 | .725 | .929 | .000 |
| Right postcentral | -0.014 | 0.01 | -1.14 | .255 | .693 | .000 |
| Right posterior cingulate | -0.032 | 0.01 | -2.65 | .008 | .203 | .001 |
| Right precentral | -0.031 | 0.01 | -2.50 | .012 | .203 | .001 |
| Right precuneus | 0.005 | 0.01 | 0.38 | .704 | .929 | .000 |
| Right rostral anterior cingulate | 0.008 | 0.01 | 0.73 | .466 | .809 | .000 |
| Right rostral middle frontal | 0.003 | 0.01 | 0.21 | .836 | .929 | .000 |
| Right superior frontal | -0.019 | 0.01 | -1.42 | .155 | .613 | .000 |
| Right superior parietal | 0.017 | 0.01 | 1.41 | .158 | .613 | .000 |
| Right superior temporal | -0.026 | 0.01 | -1.99 | .047 | .409 | .001 |
| Right supramarginal | -0.007 | 0.01 | -0.61 | .541 | .829 | .000 |
| Right frontal pole | 0.002 | 0.01 | 0.20 | .842 | .929 | .000 |
| Right temporal pole | 0.021 | 0.01 | 1.93 | .053 | .419 | .000 |
| Right transverse temporal | -0.011 | 0.01 | -0.94 | .349 | .731 | .000 |
| Right insula | 0.006 | 0.01 | 0.49 | .625 | .891 | .000 |
| Left cerebellum cortex | -0.001 | 0.01 | -0.07 | .943 | .954 | .000 |
| Left thalamus proper | -0.011 | 0.01 | -0.76 | .449 | .797 | .000 |
| Left caudate | 0.017 | 0.01 | 1.38 | .169 | .613 | .000 |
| Left putamen | 0.016 | 0.01 | 1.31 | .189 | .632 | .000 |
| Left pallidum | 0.013 | 0.01 | 1.10 | .270 | .696 | .000 |
| Brain stem | 0.008 | 0.01 | 0.59 | .553 | .829 | .000 |
| Left hippocampus | -0.011 | 0.01 | -0.84 | .399 | .748 | .000 |
| Left amygdala | 0.012 | 0.01 | 0.89 | .373 | .738 | .000 |
| Left accumbens area | 0.011 | 0.01 | 0.91 | .365 | .738 | .000 |
| Left ventral diencephalon | -0.011 | 0.02 | -0.68 | .494 | .827 | .000 |
| Right cerebellum cortex | -0.004 | 0.01 | -0.31 | .754 | .929 | .000 |
| Right thalamus proper | -0.002 | 0.02 | -0.15 | .883 | .929 | .000 |
| Right caudate | 0.027 | 0.01 | 2.20 | .028 | .304 | .001 |
| Right putamen | 0.020 | 0.01 | 1.65 | .099 | .507 | .000 |
| Right pallidum | 0.003 | 0.01 | 0.24 | .810 | .929 | .000 |
| Right hippocampus | -0.008 | 0.01 | -0.60 | .546 | .829 | .000 |
| Right amygdala | -0.002 | 0.01 | -0.16 | .871 | .929 | .000 |
| Right accumbens area | -0.002 | 0.01 | -0.21 | .835 | .929 | .000 |
| Right ventral diencephalon | -0.012 | 0.02 | -0.79 | .429 | .778 | .000 |

# **Supplementary Table 5**

## *Gray matter volume in 68 cortical and 19 subcortical regions as predictors of irritability at the third-year follow-up*

|  | *β* | *SE* | *z* | *p* | *p_fdr_* | *R^2^* |
| --- | --- | --- | --- | --- | --- | --- |
| Left banks of superior temporal sulcus | -0.001 | 0.01 | -0.08 | .938 | .949 | .000 |
| Left caudal anterior cingulate | 0.013 | 0.01 | 0.92 | .358 | .882 | .000 |
| Left caudal middle frontal | -0.005 | 0.02 | -0.34 | .736 | .882 | .000 |
| Left cuneus | 0.001 | 0.01 | 0.08 | .933 | .949 | .000 |
| Left entorhinal | -0.010 | 0.01 | -0.75 | .457 | .882 | .000 |
| Left fusiform | -0.002 | 0.02 | -0.12 | .902 | .945 | .000 |
| Left inferior parietal | -0.020 | 0.02 | -1.32 | .186 | .770 | .000 |
| Left inferior temporal | -0.010 | 0.02 | -0.58 | .562 | .882 | .000 |
| Left isthmus cingulate | 0.008 | 0.02 | 0.51 | .609 | .882 | .000 |
| Left lateral occipital | -0.005 | 0.02 | -0.29 | .769 | .882 | .000 |
| Left lateral orbitofrontal | -0.002 | 0.02 | -0.11 | .915 | .948 | .000 |
| Left lingual | -0.017 | 0.02 | -1.18 | .239 | .770 | .000 |
| Left medial orbitofrontal | -0.023 | 0.02 | -1.38 | .166 | .770 | .001 |
| Left middle temporal | -0.001 | 0.02 | -0.04 | .967 | .967 | .000 |
| Left parahippocampal | -0.010 | 0.01 | -0.72 | .471 | .882 | .000 |
| Left paracentral | -0.032 | 0.02 | -2.16 | .031 | .461 | .001 |
| Left pars opercularis | 0.006 | 0.02 | 0.43 | .668 | .882 | .000 |
| Left pars orbitalis | 0.014 | 0.01 | 0.98 | .328 | .857 | .000 |
| Left pars triangularis | 0.006 | 0.01 | 0.42 | .672 | .882 | .000 |
| Left pericalcarine | -0.009 | 0.01 | -0.68 | .498 | .882 | .000 |
| Left postcentral | -0.020 | 0.02 | -1.24 | .214 | .770 | .000 |
| Left posterior cingulate | -0.007 | 0.02 | -0.42 | .671 | .882 | .000 |
| Left precentral | -0.007 | 0.02 | -0.43 | .671 | .882 | .000 |
| Left precuneus | 0.018 | 0.02 | 1.08 | .279 | .814 | .000 |
| Left rostral anterior cingulate | 0.017 | 0.02 | 1.08 | .282 | .814 | .000 |
| Left rostral middle frontal | -0.017 | 0.02 | -1.06 | .290 | .814 | .000 |
| Left superior frontal | -0.039 | 0.02 | -2.22 | .026 | .461 | .002 |
| Left superior parietal | 0.019 | 0.02 | 1.22 | .222 | .770 | .000 |
| Left superior temporal | -0.017 | 0.02 | -1.02 | .309 | .840 | .000 |
| Left supramarginal | -0.007 | 0.02 | -0.42 | .673 | .882 | .000 |
| Left frontal pole | -0.022 | 0.01 | -1.55 | .121 | .770 | .000 |
| Left temporal pole | -0.010 | 0.01 | -0.71 | .476 | .882 | .000 |
| Left transverse temporal | -0.004 | 0.01 | -0.26 | .798 | .882 | .000 |
| Left insula | -0.005 | 0.02 | -0.32 | .749 | .882 | .000 |
| Right banks of superior temporal sulcus | -0.004 | 0.02 | -0.25 | .800 | .882 | .000 |
| Right caudal anterior cingulate | -0.030 | 0.01 | -2.10 | .036 | .461 | .001 |
| Right caudal middle frontal | 0.021 | 0.02 | 1.43 | .154 | .770 | .000 |
| Right cuneus | 0.008 | 0.02 | 0.52 | .605 | .882 | .000 |
| Right entorhinal | -0.025 | 0.01 | -1.88 | .060 | .475 | .001 |
| Right fusiform | -0.012 | 0.02 | -0.71 | .479 | .882 | .000 |
| Right inferior parietal | -0.026 | 0.02 | -1.62 | .106 | .768 | .001 |
| Right inferior temporal | 0.009 | 0.02 | 0.56 | .574 | .882 | .000 |
| Right isthmus cingulate | 0.010 | 0.02 | 0.65 | .516 | .882 | .000 |
| Right lateral occipital | 0.004 | 0.02 | 0.26 | .798 | .882 | .000 |
| Right lateral orbitofrontal | -0.007 | 0.02 | -0.41 | .681 | .882 | .000 |
| Right lingual | 0.010 | 0.01 | 0.69 | .493 | .882 | .000 |
| Right medial orbitofrontal | -0.014 | 0.02 | -0.81 | .416 | .882 | .000 |
| Right middle temporal | 0.003 | 0.02 | 0.16 | .875 | .940 | .000 |
| Right parahippocampal | -0.018 | 0.01 | -1.28 | .199 | .770 | .000 |
| Right paracentral | -0.012 | 0.02 | -0.82 | .412 | .882 | .000 |
| Right pars opercularis | 0.006 | 0.01 | 0.42 | .676 | .882 | .000 |
| Right pars orbitalis | -0.012 | 0.01 | -0.82 | .410 | .882 | .000 |
| Right pars triangularis | 0.005 | 0.01 | 0.34 | .737 | .882 | .000 |
| Right pericalcarine | 0.002 | 0.01 | 0.12 | .902 | .945 | .000 |
| Right postcentral | -0.009 | 0.02 | -0.54 | .591 | .882 | .000 |
| Right posterior cingulate | -0.020 | 0.02 | -1.27 | .205 | .770 | .000 |
| Right precentral | -0.033 | 0.02 | -1.97 | .049 | .461 | .001 |
| Right precuneus | 0.005 | 0.02 | 0.28 | .781 | .882 | .000 |
| Right rostral anterior cingulate | -0.027 | 0.01 | -1.93 | .053 | .461 | .001 |
| Right rostral middle frontal | 0.007 | 0.02 | 0.47 | .639 | .882 | .000 |
| Right superior frontal | -0.020 | 0.02 | -1.13 | .260 | .808 | .000 |
| Right superior parietal | -0.010 | 0.02 | -0.62 | .536 | .882 | .000 |
| Right superior temporal | -0.005 | 0.02 | -0.29 | .776 | .882 | .000 |
| Right supramarginal | -0.013 | 0.02 | -0.83 | .406 | .882 | .000 |
| Right frontal pole | -0.014 | 0.01 | -0.97 | .335 | .857 | .000 |
| Right temporal pole | 0.003 | 0.01 | 0.25 | .801 | .882 | .000 |
| Right transverse temporal | -0.007 | 0.02 | -0.46 | .644 | .882 | .000 |
| Right insula | -0.005 | 0.02 | -0.29 | .772 | .882 | .000 |
| Left cerebellum cortex | 0.021 | 0.02 | 1.20 | .232 | .770 | .000 |
| Left thalamus proper | 0.041 | 0.02 | 2.22 | .026 | .461 | .002 |
| Left caudate | 0.033 | 0.02 | 2.17 | .030 | .461 | .001 |
| Left putamen | 0.008 | 0.02 | 0.53 | .597 | .882 | .000 |
| Left pallidum | 0.023 | 0.02 | 1.48 | .138 | .770 | .001 |
| Brain stem | 0.048 | 0.02 | 2.64 | .008 | .461 | .002 |
| Left hippocampus | 0.009 | 0.02 | 0.52 | .606 | .882 | .000 |
| Left amygdala | 0.005 | 0.02 | 0.27 | .786 | .882 | .000 |
| Left accumbens area | 0.013 | 0.02 | 0.86 | .391 | .882 | .000 |
| Left ventral diencephalon | 0.027 | 0.02 | 1.33 | .185 | .770 | .001 |
| Right cerebellum cortex | 0.026 | 0.02 | 1.45 | .147 | .770 | .001 |
| Right thalamus proper | 0.038 | 0.02 | 1.98 | .047 | .461 | .001 |
| Right caudate | 0.032 | 0.02 | 2.03 | .042 | .461 | .001 |
| Right putamen | 0.011 | 0.02 | 0.71 | .480 | .882 | .000 |
| Right pallidum | 0.019 | 0.02 | 1.19 | .233 | .770 | .000 |
| Right hippocampus | -0.013 | 0.02 | -0.74 | .461 | .882 | .000 |
| Right amygdala | -0.004 | 0.02 | -0.23 | .819 | .891 | .000 |
| Right accumbens area | 0.006 | 0.02 | 0.42 | .677 | .882 | .000 |
| Right ventral diencephalon | 0.031 | 0.02 | 1.54 | .124 | .770 | .001 |

# **Supplementary Table 6**

## *Associations between irritability and cortical thickness in 68 cortical regions*

|  | *β* | *SE* | *z* | *p* | *p_fdr_* | *R^2^* |
| --- | --- | --- | --- | --- | --- | --- |
| Left banks of superior temporal sulcus | -0.029 | 0.02 | -1.81 | .071 | .322 | .001 |
| Left caudal anterior cingulate | 0.027 | 0.01 | 1.87 | .061 | .296 | .001 |
| Left caudal middle frontal | 0.016 | 0.02 | 0.96 | .339 | .720 | .000 |
| Left cuneus | 0.006 | 0.02 | 0.37 | .715 | .917 | .000 |
| Left entorhinal | -0.011 | 0.01 | -0.80 | .423 | .833 | .000 |
| Left fusiform | -0.001 | 0.02 | -0.05 | .964 | .985 | .000 |
| Left inferior parietal | -0.017 | 0.02 | -0.89 | .376 | .775 | .000 |
| Left inferior temporal | -0.017 | 0.02 | -1.01 | .314 | .689 | .000 |
| Left isthmus cingulate | 0.029 | 0.01 | 1.99 | .047 | .296 | .001 |
| Left lateral occipital | -0.047 | 0.02 | -2.29 | .022 | .296 | .002 |
| Left lateral orbitofrontal | -0.022 | 0.02 | -1.33 | .184 | .521 | .000 |
| Left lingual | -0.019 | 0.02 | -1.12 | .262 | .636 | .000 |
| Left medial orbitofrontal | -0.030 | 0.02 | -2.00 | .045 | .296 | .001 |
| Left middle temporal | -0.007 | 0.02 | -0.40 | .687 | .917 | .000 |
| Left parahippocampal | 0.001 | 0.02 | 0.06 | .954 | .985 | .000 |
| Left paracentral | -0.028 | 0.02 | -1.63 | .102 | .434 | .001 |
| Left pars opercularis | 0.010 | 0.02 | 0.60 | .546 | .870 | .000 |
| Left pars orbitalis | 0.008 | 0.02 | 0.53 | .599 | .905 | .000 |
| Left pars triangularis | 0.021 | 0.02 | 1.30 | .193 | .525 | .000 |
| Left pericalcarine | 0.009 | 0.02 | 0.60 | .550 | .870 | .000 |
| Left postcentral | -0.036 | 0.02 | -2.15 | .032 | .296 | .001 |
| Left posterior cingulate | 0.033 | 0.02 | 2.22 | .027 | .296 | .001 |
| Left precentral | -0.048 | 0.02 | -2.87 | .004 | .272 | .002 |
| Left precuneus | -0.005 | 0.02 | -0.25 | .801 | .985 | .000 |
| Left rostral anterior cingulate | 0.028 | 0.02 | 1.88 | .061 | .296 | .001 |
| Left rostral middle frontal | 0.013 | 0.02 | 0.65 | .515 | .870 | .000 |
| Left superior frontal | 0.046 | 0.02 | 2.37 | .018 | .296 | .002 |
| Left superior parietal | -0.013 | 0.02 | -0.66 | .513 | .870 | .000 |
| Left superior temporal | -0.011 | 0.02 | -0.60 | .549 | .870 | .000 |
| Left supramarginal | -0.015 | 0.02 | -0.79 | .429 | .833 | .000 |
| Left frontal pole | 0.015 | 0.02 | 1.05 | .296 | .671 | .000 |
| Left temporal pole | -0.021 | 0.02 | -1.40 | .162 | .506 | .000 |
| Left transverse temporal | 0.011 | 0.02 | 0.66 | .507 | .870 | .000 |
| Left insula | 0.005 | 0.02 | 0.29 | .770 | .970 | .000 |
| Right banks of superior temporal sulcus | -0.004 | 0.02 | -0.24 | .814 | .985 | .000 |
| Right caudal anterior cingulate | -0.003 | 0.01 | -0.19 | .852 | .985 | .000 |
| Right caudal middle frontal | 0.000 | 0.02 | -0.03 | .977 | .985 | .000 |
| Right cuneus | -0.006 | 0.02 | -0.39 | .697 | .917 | .000 |
| Right entorhinal | 0.001 | 0.01 | 0.05 | .962 | .985 | .000 |
| Right fusiform | -0.007 | 0.02 | -0.39 | .696 | .917 | .000 |
| Right inferior parietal | -0.008 | 0.02 | -0.37 | .708 | .917 | .000 |
| Right inferior temporal | -0.020 | 0.02 | -1.10 | .272 | .638 | .000 |
| Right isthmus cingulate | 0.026 | 0.01 | 1.87 | .061 | .296 | .001 |
| Right lateral occipital | -0.032 | 0.02 | -1.52 | .129 | .439 | .001 |
| Right lateral orbitofrontal | 0.003 | 0.02 | 0.17 | .865 | .985 | .000 |
| Right lingual | -0.032 | 0.02 | -1.89 | .059 | .296 | .001 |
| Right medial orbitofrontal | 0.002 | 0.02 | 0.12 | .906 | .985 | .000 |
| Right middle temporal | 0.008 | 0.02 | 0.44 | .662 | .917 | .000 |
| Right parahippocampal | -0.012 | 0.02 | -0.75 | .453 | .833 | .000 |
| Right paracentral | -0.026 | 0.02 | -1.55 | .122 | .439 | .001 |
| Right pars opercularis | 0.026 | 0.02 | 1.55 | .121 | .439 | .001 |
| Right pars orbitalis | 0.022 | 0.02 | 1.38 | .169 | .506 | .000 |
| Right pars triangularis | 0.036 | 0.02 | 2.19 | .028 | .296 | .001 |
| Right pericalcarine | -0.012 | 0.02 | -0.77 | .444 | .833 | .000 |
| Right postcentral | -0.024 | 0.02 | -1.53 | .127 | .439 | .001 |
| Right posterior cingulate | 0.000 | 0.01 | -0.02 | .985 | .985 | .000 |
| Right precentral | -0.019 | 0.02 | -1.25 | .210 | .549 | .000 |
| Right precuneus | -0.002 | 0.02 | -0.10 | .920 | .985 | .000 |
| Right rostral anterior cingulate | 0.020 | 0.01 | 1.37 | .171 | .506 | .000 |
| Right rostral middle frontal | 0.039 | 0.02 | 2.11 | .035 | .296 | .002 |
| Right superior frontal | 0.044 | 0.02 | 2.22 | .027 | .296 | .002 |
| Right superior parietal | -0.008 | 0.02 | -0.42 | .672 | .917 | .000 |
| Right superior temporal | -0.002 | 0.02 | -0.12 | .905 | .985 | .000 |
| Right supramarginal | 0.007 | 0.02 | 0.37 | .712 | .917 | .000 |
| Right frontal pole | 0.002 | 0.02 | 0.14 | .892 | .985 | .000 |
| Right temporal pole | 0.009 | 0.02 | 0.57 | .567 | .876 | .000 |
| Right transverse temporal | 0.003 | 0.02 | 0.22 | .827 | .985 | .000 |
| Right insula | -0.018 | 0.02 | -1.18 | .237 | .597 | .000 |

# **Supplementary Table 7**

## *Associations between irritability and surface area in 68 cortical regions*

|  | *β* | *SE* | *z* | *p* | *p_fdr_* | *R^2^* |
| --- | --- | --- | --- | --- | --- | --- |
| Left banks of superior temporal sulcus | -0.001 | 0.02 | -0.07 | .946 | .989 | .000 |
| Left caudal anterior cingulate | -0.009 | 0.02 | -0.60 | .547 | .827 | .000 |
| Left caudal middle frontal | 0.027 | 0.02 | 1.54 | .124 | .524 | .001 |
| Left cuneus | 0.022 | 0.02 | 1.39 | .165 | .561 | .000 |
| Left entorhinal | -0.001 | 0.01 | -0.08 | .935 | .989 | .000 |
| Left fusiform | -0.017 | 0.02 | -0.85 | .396 | .702 | .000 |
| Left inferior parietal | -0.018 | 0.02 | -1.02 | .307 | .702 | .000 |
| Left inferior temporal | 0.009 | 0.02 | 0.44 | .661 | .917 | .000 |
| Left isthmus cingulate | -0.008 | 0.02 | -0.44 | .658 | .917 | .000 |
| Left lateral occipital | 0.017 | 0.02 | 0.94 | .345 | .702 | .000 |
| Left lateral orbitofrontal | 0.023 | 0.02 | 1.10 | .272 | .702 | .001 |
| Left lingual | 0.025 | 0.02 | 1.64 | .101 | .505 | .001 |
| Left medial orbitofrontal | 0.051 | 0.02 | 2.71 | .007 | .159 | .003 |
| Left middle temporal | 0.008 | 0.02 | 0.37 | .712 | .922 | .000 |
| Left parahippocampal | 0.013 | 0.02 | 0.82 | .410 | .702 | .000 |
| Left paracentral | 0.015 | 0.02 | 0.90 | .366 | .702 | .000 |
| Left pars opercularis | 0.013 | 0.02 | 0.82 | .413 | .702 | .000 |
| Left pars orbitalis | -0.005 | 0.02 | -0.28 | .780 | .924 | .000 |
| Left pars triangularis | 0.010 | 0.02 | 0.65 | .517 | .827 | .000 |
| Left pericalcarine | 0.025 | 0.01 | 1.78 | .076 | .431 | .001 |
| Left postcentral | -0.001 | 0.02 | -0.07 | .948 | .989 | .000 |
| Left posterior cingulate | -0.028 | 0.02 | -1.62 | .104 | .505 | .001 |
| Left precentral | -0.012 | 0.02 | -0.62 | .533 | .827 | .000 |
| Left precuneus | 0.007 | 0.02 | 0.37 | .713 | .922 | .000 |
| Left rostral anterior cingulate | -0.006 | 0.02 | -0.33 | .742 | .922 | .000 |
| Left rostral middle frontal | -0.010 | 0.02 | -0.51 | .611 | .884 | .000 |
| Left superior frontal | -0.008 | 0.02 | -0.35 | .723 | .922 | .000 |
| Left superior parietal | 0.023 | 0.02 | 1.24 | .216 | .668 | .001 |
| Left superior temporal | -0.045 | 0.02 | -2.19 | .028 | .282 | .002 |
| Left supramarginal | -0.035 | 0.02 | -2.04 | .042 | .313 | .001 |
| Left frontal pole | 0.016 | 0.02 | 0.95 | .344 | .702 | .000 |
| Left temporal pole | -0.015 | 0.02 | -0.87 | .382 | .702 | .000 |
| Left transverse temporal | -0.019 | 0.02 | -1.21 | .227 | .671 | .000 |
| Left insula | -0.028 | 0.02 | -1.51 | .131 | .524 | .001 |
| Right banks of superior temporal sulcus | -0.013 | 0.02 | -0.82 | .413 | .702 | .000 |
| Right caudal anterior cingulate | -0.006 | 0.02 | -0.37 | .715 | .922 | .000 |
| Right caudal middle frontal | -0.005 | 0.02 | -0.27 | .788 | .924 | .000 |
| Right cuneus | 0.029 | 0.02 | 1.78 | .075 | .431 | .001 |
| Right entorhinal | -0.014 | 0.01 | -0.96 | .336 | .702 | .000 |
| Right fusiform | 0.006 | 0.02 | 0.31 | .759 | .922 | .000 |
| Right inferior parietal | -0.036 | 0.02 | -2.00 | .046 | .313 | .001 |
| Right inferior temporal | -0.030 | 0.02 | -1.47 | .143 | .540 | .001 |
| Right isthmus cingulate | 0.011 | 0.02 | 0.68 | .496 | .823 | .000 |
| Right lateral occipital | 0.020 | 0.02 | 1.06 | .291 | .702 | .000 |
| Right lateral orbitofrontal | 0.003 | 0.02 | 0.17 | .864 | .965 | .000 |
| Right lingual | 0.039 | 0.02 | 2.52 | .012 | .204 | .002 |
| Right medial orbitofrontal | 0.001 | 0.02 | 0.03 | .977 | .989 | .000 |
| Right middle temporal | -0.064 | 0.02 | -2.89 | .004 | .136 | .004 |
| Right parahippocampal | 0.000 | 0.02 | 0.02 | .984 | .989 | .000 |
| Right paracentral | 0.005 | 0.02 | 0.31 | .758 | .922 | .000 |
| Right pars opercularis | 0.014 | 0.02 | 0.89 | .372 | .702 | .000 |
| Right pars orbitalis | 0.003 | 0.02 | 0.15 | .880 | .965 | .000 |
| Right pars triangularis | 0.014 | 0.02 | 0.90 | .368 | .702 | .000 |
| Right pericalcarine | 0.032 | 0.02 | 2.19 | .029 | .282 | .001 |
| Right postcentral | -0.020 | 0.02 | -0.99 | .324 | .702 | .000 |
| Right posterior cingulate | -0.035 | 0.02 | -2.01 | .045 | .313 | .001 |
| Right precentral | -0.043 | 0.02 | -2.20 | .028 | .282 | .002 |
| Right precuneus | 0.032 | 0.02 | 1.52 | .129 | .524 | .001 |
| Right rostral anterior cingulate | 0.022 | 0.02 | 1.34 | .180 | .583 | .000 |
| Right rostral middle frontal | 0.000 | 0.02 | -0.01 | .989 | .989 | .000 |
| Right superior frontal | -0.023 | 0.02 | -1.06 | .292 | .702 | .001 |
| Right superior parietal | 0.060 | 0.02 | 3.21 | .001 | .068 | .004 |
| Right superior temporal | -0.031 | 0.02 | -1.39 | .165 | .561 | .001 |
| Right supramarginal | -0.009 | 0.02 | -0.54 | .591 | .874 | .000 |
| Right frontal pole | -0.010 | 0.02 | -0.60 | .546 | .827 | .000 |
| Right temporal pole | 0.015 | 0.02 | 0.98 | .329 | .702 | .000 |
| Right transverse temporal | -0.003 | 0.02 | -0.15 | .880 | .965 | .000 |
| Right insula | -0.003 | 0.02 | -0.17 | .863 | .965 | .000 |


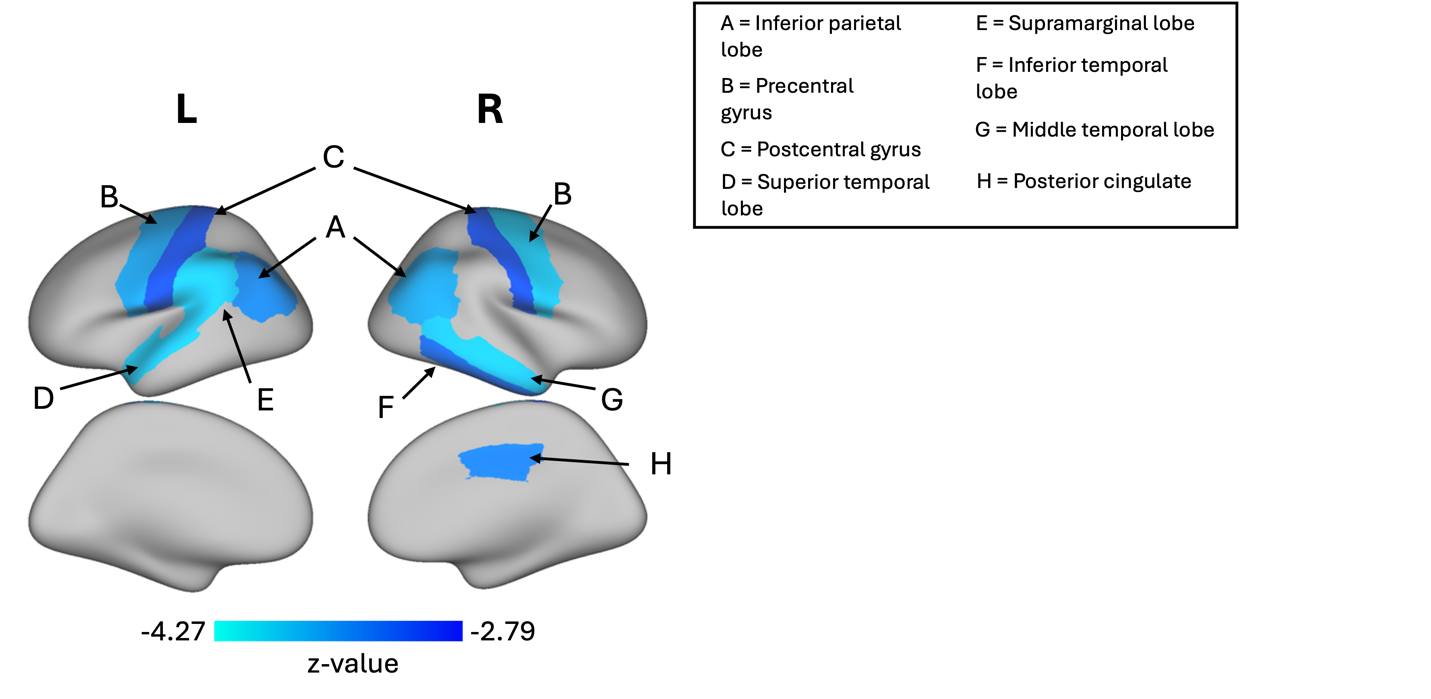


**Supplementary Figure 1.** Brain regions with significant associations with irritability (same regions as Figure 2). The figure highlights areas with smaller gray matter volume, with each region labeled for ease of identification. Details on the statistical significance of these results are provided in Supplementary Table 1.
